# Quorum-sensing regulator LsrR modulates resistance to oxidative stress by interfering with sulfate assimilation in avian pathogenic *Escherichia coli*

**DOI:** 10.1128/jb.00044-25

**Published:** 2026-06-12

**Authors:** Lanfang Kong, Chuntian Tu, Xiangjun Song, Yang Wang, Zhihao Wang, Zhaoguo Chen, Wenyan Fan, Lianhua Nie, Wei Jiang, Xiangan Han

**Affiliations:** 1 Shanghai Veterinary Research Institute, Chinese Academy of Agricultural Sciences (CAAS) 118161 , Shanghai, China; 2 College of Animal Science and Technology, Henan University of Science and Technology 74623, Luoyang, China; 3 Anhui Province Engineering Laboratory for Animal Food Quality and Bio-safety, College of Animal Science and Technology, Anhui Agricultural University 605541 , Hefei, China; University of Virginia School of Medicine, Charlottesville, Virginia, USA

**Keywords:** avian pathogenic *E. coli *(APEC), LsrR transcription factor, sulfate assimilation pathway, oxidative stress, intracellular survival

## Abstract

Prokaryotes and eukaryotes endogenously generate the gaseous molecule hydrogen sulfide (H_2_S), which has been identified as a novel signaling molecule. Bacterial H_2_S plays a protective role against reactive oxygen species and antibiotic-induced cellular damage. Endogenous H_2_S can be produced by the assimilation pathway of sulfate and thiosulfate in *Escherichia coli*. The transcription regulator LsrR influences various bacterial characteristics. The results of the present study revealed that avian pathogenic *E. coli* (APEC) lacking LsrR exhibited increased H_2_S production and enhanced tolerance to oxidative stress. Transcriptome sequencing revealed the upregulation of genes related to the sulfate assimilation pathway after the deletion of *lsrR*. Further investigation demonstrated that LsrR acts as a transcriptional repressor, binds directly to the promoter region of *cysPUWAM,* and interacts with both CysJ and CysN, thereby inhibiting exogenous sulfate assimilation and decreasing the oxidative stress resistance of APEC. Additionally, the absence of *lsrR* facilitated the survival of APEC94 in macrophage RAW264.7 cells. Our findings suggest that LsrR represses resistance to oxidative stress in APEC by interfering with the sulfate assimilation pathway and provide valuable insights into host-pathogen interactions.

**IMPORTANCE:**

Avian pathogenic *Escherichia coli* (APEC) exhibits complex serotypes and widespread antibiotic resistance, placing a significant economic burden on the poultry industry. The LuxS/AI-2 quorum-sensing systems, which are closely associated with bacterial growth, biofilm formation, and virulence, play important regulatory roles in the virulence of *E. coli* (including avian pathogenic *E. coli*, serotype O_78_) by regulating the bacterial response to diverse antibiotics. The LuxS/AI-2 system is under the transcriptional control of the LsrR repressor. Despite this, the pathophysiological function of LsrR in pathogen-host interactions remains elusive. The results of the present study revealed that LsrR plays a crucial role in modulating various biological properties of APEC. Specifically, it inhibits sulfate transport, thereby reducing both the resistance of bacteria to oxidative stress and their survival within macrophages. In summary, LsrR is involved in the regulation of key processes during pathogen-host interactions. These findings provide promising prophylactic strategies for the prevention and control of APEC infections.

## INTRODUCTION

Avian pathogenic *Escherichia coli* (APEC) is the causative agent of colibacillosis, which leads to significant economic losses in the poultry industry and poses a potential threat to public health (1, 2). Hydrogen sulfide (H_2_S) serves as a gaseous signaling molecule in both eukaryotes and prokaryotes and plays a crucial role in various intracellular and extracellular processes, particularly the oxidative stress responses (3–5). Bacteria generate H_2_S by utilizing inorganic sulfur compounds (such as sulfate and sulfite) and organic sulfur compounds (such as L-cysteine and taurine). The two primary pathways for sulfate metabolism are dissimilatory sulfate reduction and assimilatory sulfate reduction (ASR) (6–8). LsrR, a regulator of the LuxS/AI-2 type quorum-sensing system, is involved in regulating the oxidative stress response of pathogenic *E. coli* (9, 10). Studies suggest that H_2_S contributes to bacterial resistance against oxidative stress (11). However, the mechanism by which LsrR regulates H_2_S production remains elusive.

**Fig 1:**
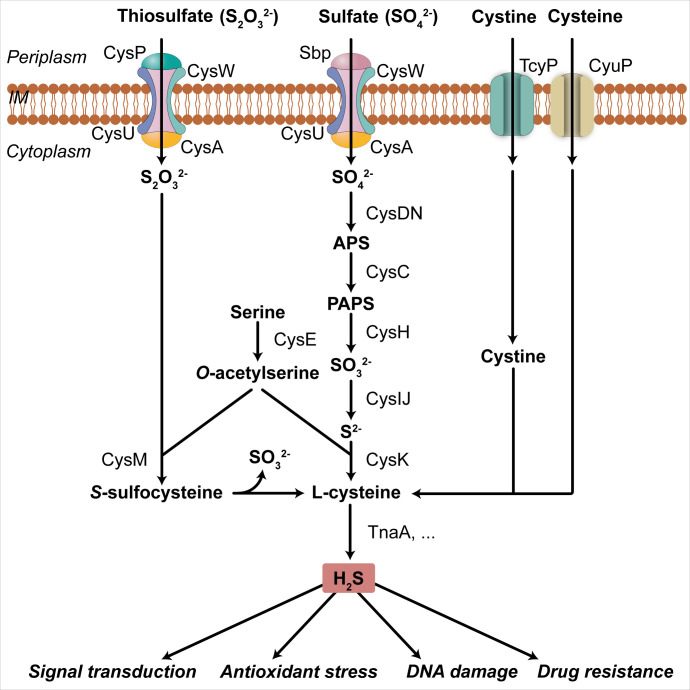
Sulfur metabolism in *Escherichia coli*. Exogenous thiosulfate and sulfate are assimilated and reduced to L-cysteine. In contrast, extracellular cystine and cysteine are imported via dedicated transporters and subsequently converted to L-cysteine, thereby supporting intracellular sulfur homeostasis and downstream metabolic processes.

Research has indicated that both endogenous H_2_S and exogenous sulfate metabolism protect pathogens from various antibiotic disturbances through distinct mechanisms (12, 13). In model bacteria such as *E. coli* and *Bacillus*, intracellular L-cysteine is synthesized through sulfate and thiosulfate assimilation, cysteine and cystine transport, and L-serine conversion (14, 15) (Fig. 1). During the cultivation of *E. coli* in Luria-Bertani (LB) broth, endogenous H_2_S is primarily produced from L-cysteine by the enzyme 3-mercaptopyruvate sulfurtransferase (3-MST) (16, 17). This enzyme safeguards *E. coli* from oxidative stress through L-cysteine metabolism and H_2_S-mediated sequestration of free iron, which is vital for the genotoxic Fenton reaction (18, 19). Therefore, pioglitazone, an inhibitor of 3-MST, can reduce H_2_S levels and increase reactive oxygen species (ROS) levels. This mechanism enables it to effectively kill pathogenic bacteria without causing damage to host cells during pathogen–host interactions (20). Additionally, YcjW, a LacI-type DNA-binding transcriptional regulator, regulates the expression of PspE (thiosulfate thiotransferase) and genes involved in carbohydrate metabolism. The expression of PspE provides another mechanism for the biosynthesis of endogenous H_2_S (21).

While earlier studies emphasized microbial H_2_S production via dissimilatory sulfite reductase, further research suggests that the gut microbiota degrades sulfopolysaccharides and assimilates sulfate to produce H_2_S through the ASR pathway, highlighting its greater significance in the human gut (22–24). Although studies have elucidated the mechanism of endogenous H_2_S and exogenous thiosulfate assimilation, the exact mechanism by which exogenous sulfate influences bacterial physiological activities remains unclear.

LsrR, the repressor of the LuxS/AI-2 system, effectively inhibits the biofilm formation ability of the mammary pathogen *Escherichia coli* (MPEC) by directly binding to the promoter region of the *fim* operon, which encodes type 1 fimbrial adhesion factor (25, 26). Furthermore, overexpression of *lsrR* downregulates the transcription levels of *ahpCF* and *katG*, which encode key enzymes responsible for scavenging excessive hydrogen peroxide in MPEC, thereby decreasing bacterial survival under H_2_O_2_ stress (10, 27). Research has shown that sulfur compounds can inhibit the expression of quorum-sensing system (QS)-related genes (*lasB*, *rhlA,* and *pqsA*) and regulate bacterial metabolism in *Pseudomonas aeruginosa* (28, 29). However, the role of *lsrR* in mediating sulfate assimilation to combat oxidative stress remains under investigation. H_2_S functions as a bacterial signaling molecule that protects bacteria from host immunity (30, 31). Inhibiting H_2_S production increases the sensitivity of *E. coli* and *Staphylococcus aureus* to elimination by immune cells. Additionally, *E. coli* strains lacking 3-MST exhibit reduced survival in macrophage RAW264.7 cells (32, 33). Similarly, *Vibrio cholerae* regulates cystathionine-β-synthase-dependent H_2_S production to increase survival and proliferation ability under oxidative stress (34–36). Despite these findings, the mechanism by which *lsrR* influences host-pathogen interactions in APEC remains unclear.

Given the crucial role of endogenous H_2_S production in the bacterial antioxidant emergency response, it is regarded as a potential target for antimicrobial therapy. However, as a key step in designing specific inhibitors that suppress H_2_S production, it is necessary to explore alternative sources of endogenous H_2_S production. The present study pursued this goal by elucidating the mechanisms by which LsrR interferes with APEC sulfate assimilation, the antioxidant emergency response, and host cell survival. This research provides novel insights into the prevention and control of APEC infections.

## RESULTS

### LsrR modulates the biological characteristics of *E. coli*

**Fig 2:**
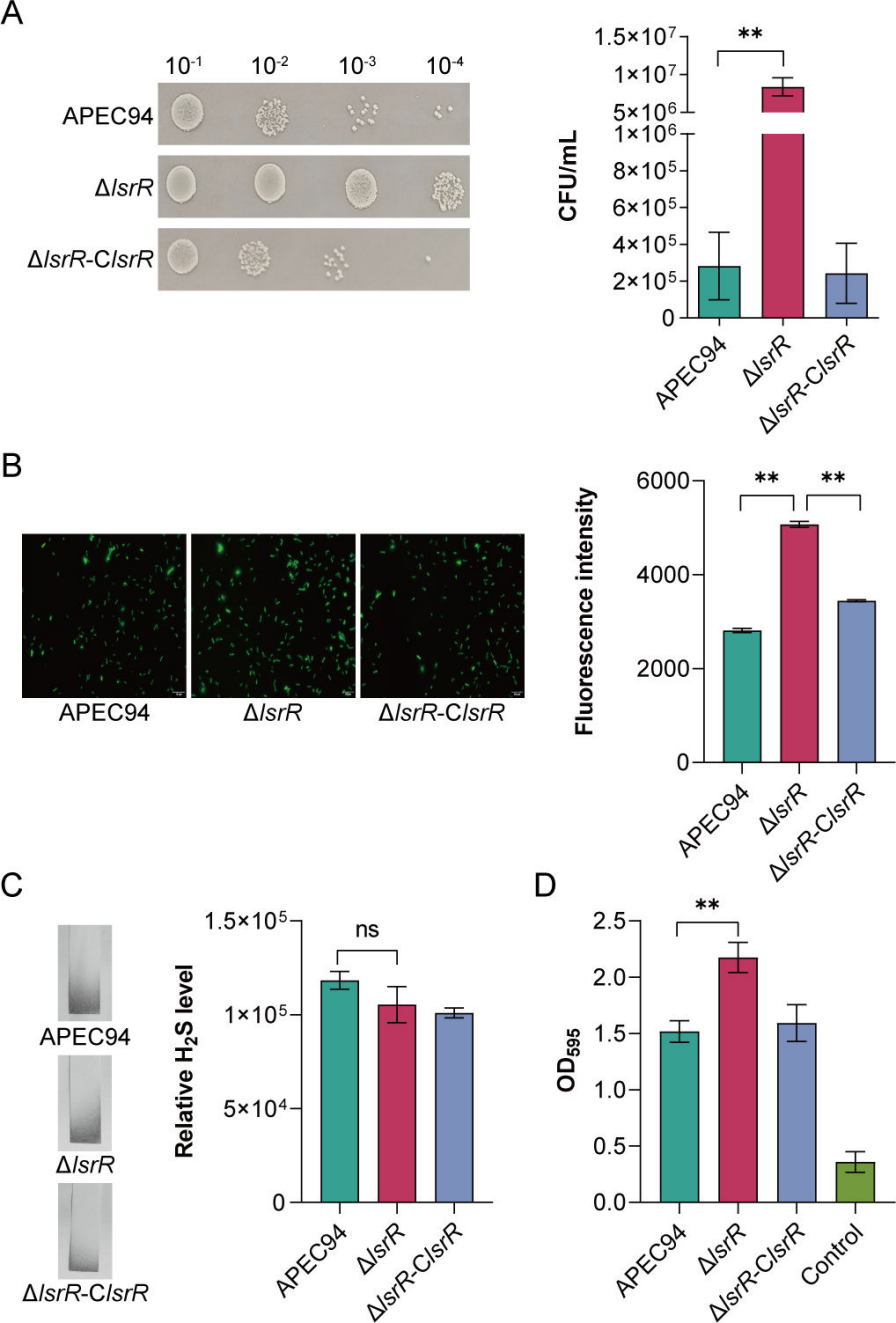
Detection of antioxidant stress ability, H_2_S production, and biofilm formation in the *lsrR* mutant strain. (**A**) Resistance to H_2_O_2_. Wild-type, *lsrR* mutant, and complementary strains (OD_600_ = 1.0) were cultured in 5 mL of LB supplemented with 8 μL of H_2_O_2_ (30%) at 37°C for 1 h. Three microliters of each dilution was spotted on LB agar plates and incubated at 37°C for 12 h (left panel). The number of colony-forming units (CFUs) of each strain was determined using the dilution method (right panel). (**B, C**) Determination of extracellular or intracellular H_2_S production. Intracellular H_2_S levels were determined using a WSP-5 probe (**B**). Extracellular H_2_S levels were determined using lead acetate test paper (**C**). For the lead acetate test, the strips were suspended above the liquid medium and cultured for 24 h. Hydrogen sulfide was captured by the strips and turned black. Grayscale values were measured to compare extracellular H_2_S production. For intracellular H_2_S detection, each strain was cultured (OD_600_ = 1.0), supplemented with the WSP-5 probe at a final concentration of 15 μM, and then incubated at 37°C for 30 min. The bacteria were resuspended in PBS, and the fluorescence intensity at 488–528 nm (FITC) was measured using a Cytation3 (BioTek). (**D**) Detection of biofilm formation by crystal violet staining. The absorbance at 595 nm was measured. Data are presented as the mean ± standard deviation (SD) from at least three independent experiments. The blank control was a Luria-Bertani-cultured well incubated under identical conditions but receiving no bacterial inoculum. Student’s *t*-test was used for statistical analysis, with significance indicated as ns (no significant change, *P* > 0.05), *(0.01 < *P* < 0.05), and **(*P* < 0.01).

QS regulates various bacterial traits. Here, we hypothesized that the activity of LsrR, a QS-associated transcriptional regulator, influences *E. coli* physiology. The *lsrR*-deficient mutant exhibited increased H_2_O_2_ tolerance (*P* < 0.01) (Fig. 2A). Since H_2_S contributes to oxidative stress resistance, we quantified the intracellular and extracellular H_2_S levels. Analysis of lead acetate paper and the WSP-5 probe revealed a significant increase in intracellular H_2_S in the APEC94Δ*lsrR* mutant (*P* < 0.01) (Fig. 2B), whereas extracellular H_2_S remained unchanged (*P* > 0.05) (Fig. 2C). Additionally, biofilm formation was significantly enhanced in the APEC94Δ*lsrR* strain at 25°C (*P* < 0.01) (Fig. 2D) and 37°C (*P* < 0.05) (Fig. S1). These findings indicate that *lsrR* deletion elevates intracellular H_2_S and enhances the oxidative stress resistance of APEC.

### LsrR regulates the transcription of genes involved in sulfate assimilation

**Fig 3:**
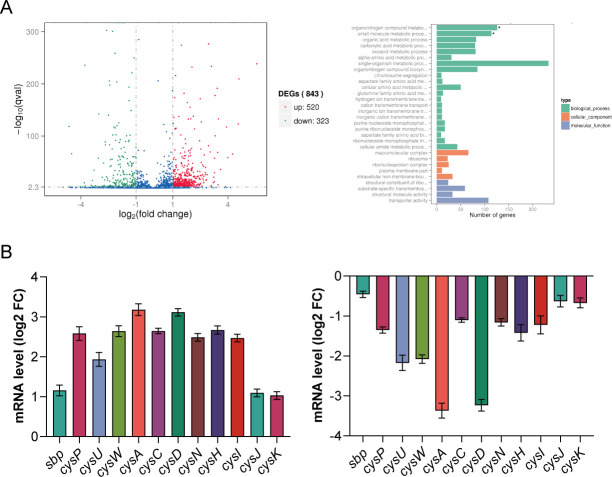
Transcriptomic analysis and RT-qPCR were used to measure the mRNA levels of genes related to sulfur metabolism. (**A**) Transcriptomic analysis of the transcriptional changes caused by the deletion of *lsrR*. Differentially expressed genes in the Δ*lsrR* strain (left panel) and the number of genes per category (right panel). (**B**) RT-qPCR detection of the mRNA levels of sulfur metabolism-related genes in the Δ*lsrR* strain (left panel) and the *lsrR*-overexpressing wild-type strain (right panel). RNA was extracted from the WT, Δ*lsrR,* and *lsrR* (overexpression) strains using TRIzol. Real-time qPCR was performed using the SYBR qPCR Kit, and the 2^-ΔΔCt^ method was used to calculate the fold change. The mRNA levels in both the left and right panels were quantified relative to those in the wild-type strain. Data are presented as the mean ± SD from at least three independent experiments.

**Fig 4:**
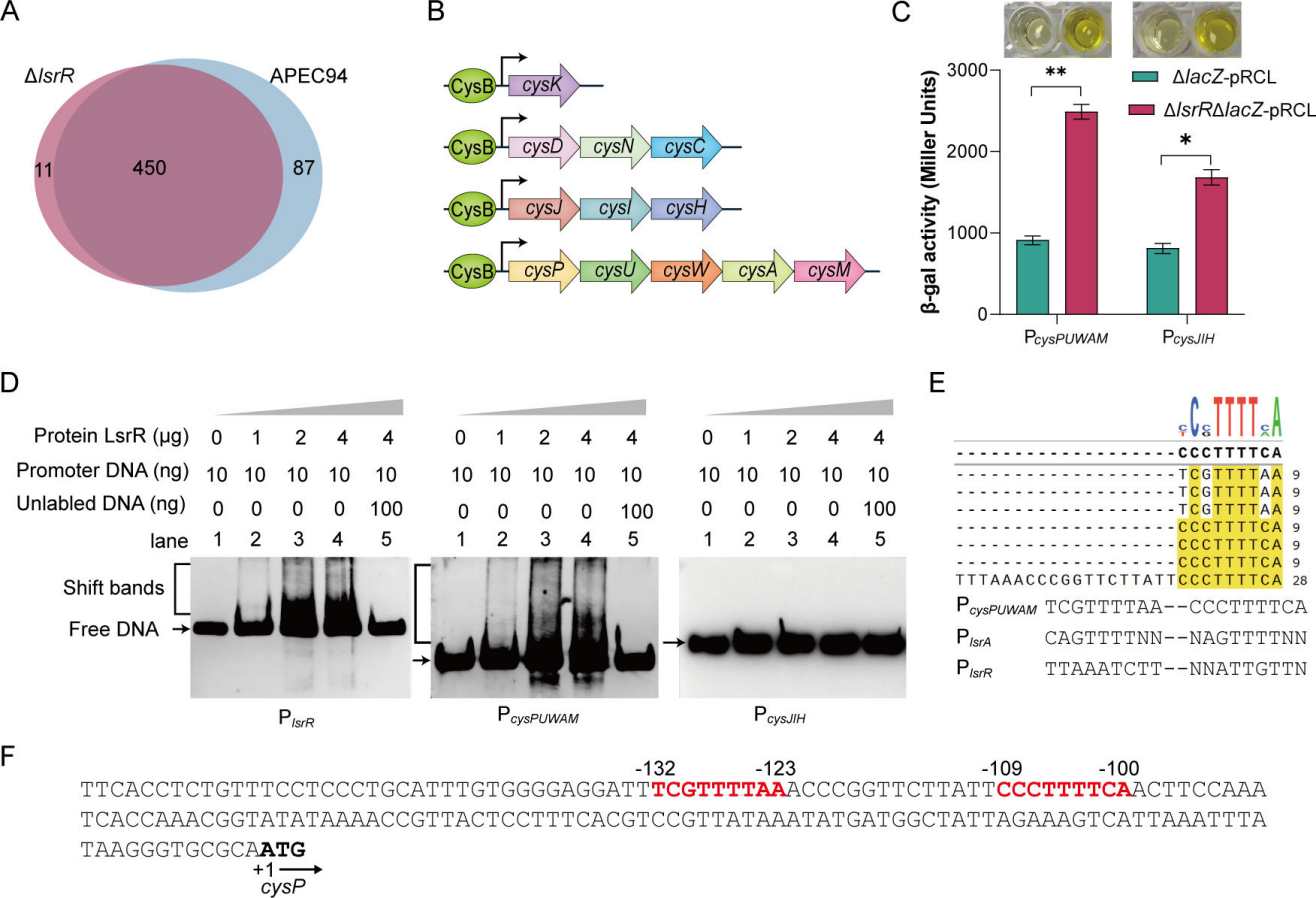
Chromatin immunoprecipitation sequencing (ChIP-seq) and electrophoretic mobility shift assay (EMSA) for the detection of the binding of LsrR to *cysPUWAM* promoter DNA. (**A**) Venn diagram for analysis of ChIP-seq data. (**B**) Diagrams of the *cys* loci. Data was obtained from the NCBI genome database. (**C**) Promoter activity detected by a β-galactosidase assay. pRCL plasmids fused with the promoter DNA of *cysPUWAM* and *cysJIH* were transformed into the Δ*lacZ* or Δ*lacZ*Δ*lsrR* strain to detect β-galactosidase activity. The cells were disrupted by ultrasonication, and 20 μL of the supernatant and 5 μg of o-nitrophenyl-β-D-galactopyranoside were used to detect β-galactosidase activity. Galactosidase activity was calculated using the Miller unit formula. Data are presented as the mean ± SD from at least three independent experiments. Student’s *t*-test was used for statistical analysis, with significance indicated as ns (no significant change, *P* > 0.05), *(0.01 < *P* < 0.05), and **(*P* < 0.01). (**D**) EMSA was performed to verify the binding of LsrR to the promoter regions of the *cysPUWAM* and *cysJIH* genes. LsrR-His protein was purified with nickel-tagged resin. After incubation at 37°C for 30 min, protein-DNA complexes were analyzed by 6% nondenaturing gel electrophoresis and transferred onto a nylon membrane. Images were captured using horseradish peroxidase (HRP). (**E**) A DNase I footprinting assay verified the binding sites of the cys*PUWAM* promoter. (**F**) Identification of binding sites within the *cysPUWAM* promoter through sequencing analysis following DNase I footprinting.

To investigate the mechanism underlying the increase in H_2_S production in the Δ*lsrR* strain, RNA-seq was performed. Deletion of *lsrR* led to the upregulation of 520 genes and the downregulation of 323 genes (Fig. 3A). Gene ontology analysis revealed significant enrichment in small-molecule metabolic processes (Fig. 3A). Notably, exogenous H_2_S production has been linked to the inorganic sulfur assimilation pathway. It has been reported that *sbp*, *cysP*, and *cysUWA* serve as transporters for sulfate and thiosulfate. Additionally, the genes *cysD*, *cysN*, *cysC*, *cysH*, *cysIJ*, *cysM*, and *cysK* are involved in the assimilation of exogenous sulfate and thiosulfate (Fig. 4B). Real-time quantitative PCR (RT-qPCR) results demonstrated that deletion of *lsrR* led to the upregulation of genes involved in sulfate assimilation (Fig. 3B). However, no significant change in the transcriptional level of the *cysM* gene in the thiosulfate assimilation pathway was detected (Table S3).

To confirm the role of *lsrR* in sulfate assimilation, we constructed an *lsrR* overexpression system in APEC with a pBAD33 plasmid induced by L-arabinose. Overexpressing *lsrR* downregulated sulfate assimilation genes (Fig. 3B), highlighting its regulatory role in this pathway.

### LsrR binds to the promoters of sulfate transport genes

As a transcriptional regulator, LsrR contains a DNA-binding domain. To identify its direct targets, we performed chromatin immunoprecipitation sequencing (ChIP-seq), which revealed enrichment of 87 genes (Fig. 4A). Notably, these differentially expressed genes are involved in sulfate assimilation. We hypothesize that LsrR regulates their transcription by binding to their promoters. A schematic of these genes and their promoters is shown in Fig. 4B.

The deletion of *lsrR* resulted in increased transcription from the *cysPUWAM* and *cysJIH* promoters as measured through β-galactosidase activity (Fig. 4C). To confirm the binding of LsrR, biotin-labeled *cysPUWAM, cysJIH,* and *lsrR* promoters were used in an electrophoretic mobility shift assay (EMSA). With increasing concentrations of the LsrR protein, shifted bands appeared, indicating that LsrR binds to the *cysPUWAM* and *lsrR* promoters. However, LsrR does not bind to the *cysJIH* promoter (Fig. 4D). The *lsrR* promoter served as a positive control. The unlabeled versions of each promoter were used as competitors.

DNase I footprinting confirmed that LsrR binds to *cysPUWAM* promoter DNA. Considering that LsrR binds to the *lsrA* and *lsrR* promoter DNA, the promoter sequences of *cysPUWAM*, *lsrA,* and *lsrR* were aligned to identify conserved motifs. Sequence alignment revealed an 18 nt region, with the conserved motif NNNTTTTNN--NNNTTTTNN, as the binding site for LsrR (Fig. 4E). Through sequencing analysis, LsrR was shown to bind to regions of the *cysPUWAM* promoter from −132 bp to −123 bp and from −109 bp to −100 bp (Fig. 4F).

### LsrR interacts with CysJ and CysN

**Fig 5:**
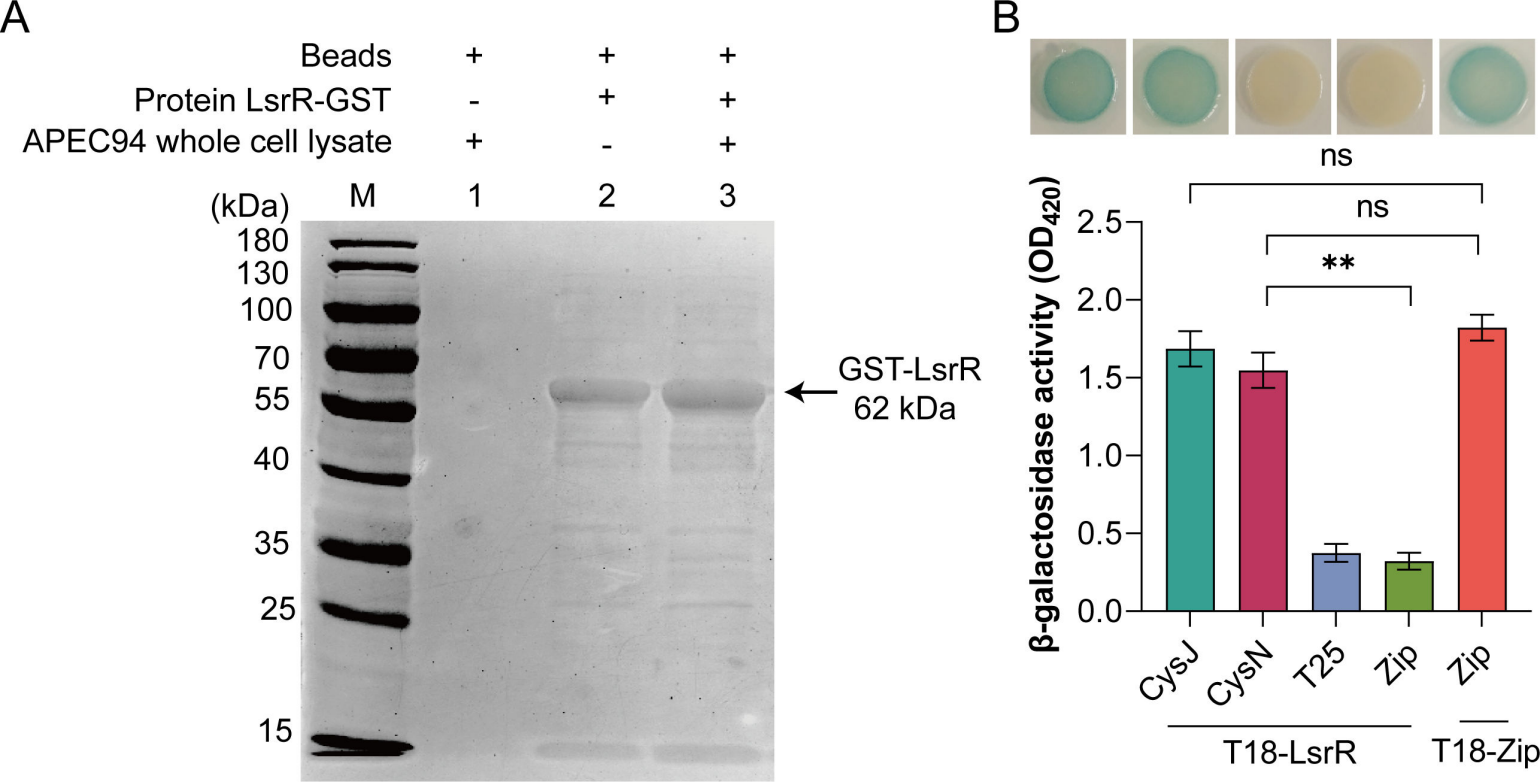
GST pull-down and bacterial adenylate cyclase two-hybrid (BACTH) assays for screening proteins that interact with LsrR. (**A**) The LsrR-GST fusion protein was incubated with or without APEC94 whole-cell lysate at 4°C for 12 h. Soluble LsrR-GST protein was bound to GSH-labeled magnetic beads, forming LsrR-GST-GSH complexes (lane 2), or was incubated with APEC94 whole-cell lysate for protein–protein interaction analysis (lane 3). Samples without the LsrR-GST protein were used as negative controls (lane 1). (**B**) BACTH assay to verify interacting proteins. LsrR was inserted into pUT18c, while CysJ and CysN were inserted into pKT25. Both plasmids were cotransformed into *E. coli* BTH101. BTH101 was cultured on LB agar plates supplemented with 0.5 mM IPTG and 40 μg/mL X-Gal at 30°C. Zip-Zip was used as a positive control. The empty pKT25 or pKT25-Zip plasmid was used as a blank or a negative control. Data are presented as the mean ± SD from at least three independent experiments. Student’s *t*-test was used for statistical analysis, with significance indicated as ns (no significant change, *P* > 0.05), *(0.01 < *P* < 0.05), and **(*P* < 0.01).

Previous research has demonstrated that LsrR binds to the promoter DNA region and regulates the expression of the exogenous sulfate transporter gene *cysPUAWM*. To identify proteins that interact with LsrR, a GST pull-down assay was performed. Soluble LsrR-GST protein was bound to GSH-labeled magnetic beads, forming LsrR-GST-GSH complexes, or was incubated with APEC94 whole-cell lysate for protein–protein interaction analysis (Fig. 5A). Eluted samples obtained from the GST pull-down assay were analyzed by liquid chromatography-mass spectrometry (LC-MS), and the results revealed that LsrR interacts with CysJ and CysN (Table S5).

To validate the protein interactions identified in the GST pull-down assays, a bacterial adenylate cyclase two-hybrid (BACTH) assay was conducted. As shown in Table S2, we assessed the potential interactions of the protein LsrR with 20 proteins (Table S2, Primers for BACTH) involved in the L-cysteine biosynthesis pathway. The *lsrR* open reading frame (ORF) was inserted into pUT18c, and the *cysJ* and *cysN* ORFs were inserted into pKT25. The plasmids were subsequently transformed into the BTH101 strain. The construction of the other BTH101-interacting strains was the same as described above. Fusion constructs of LsrR-CysJ and LsrR-CysN produced blue colonies, confirming these interactions (Fig. 5B, top panel). A Zip-Zip fusion was used as a positive control, and empty plasmids were used as negative controls.

The statistical significance of the change in β-gal activity in the interactions between LsrR-CysJ and LsrR-CysN was compared with that in the negative control and other noninteracting proteins. The activity levels in the LsrR-CysJ and LsrR-CysN groups did not significantly differ from those in the positive control group (*P* > 0.05) but were significantly greater than those in the negative control group (*P* < 0.01) (Fig. 5B, bottom panel). These results confirm the interactions between LsrR and the sulfate assimilation proteins CysJ and CysN.

### Molecular docking analysis of protein-protein interactions between LsrR and CysJ and CysN

**Fig 6:**
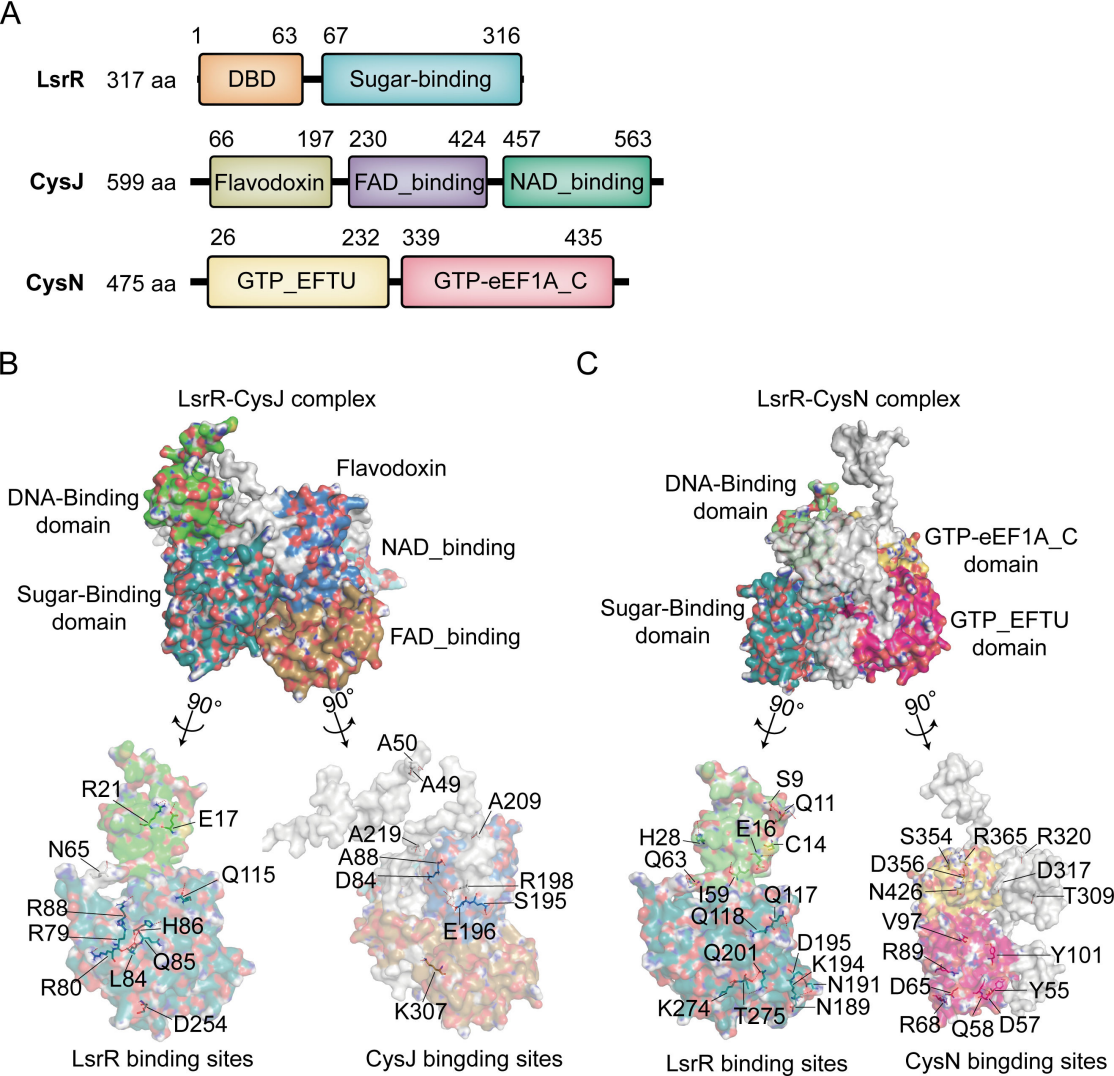
3D protein modeling and protein-protein molecular docking. (**A**) Domain architecture of three proteins based on the UniProt database (https://www.uniprot.org/). (**B**) Visualization of 3D models of the three proteins. A 3D model of CysN was generated using SWISS-MODEL (https://swissmodel.expasy.org). 3D models of LsrR (PDB ID: 3GO1) and CysJ (1YKG) were obtained from the Protein Data Bank (https://www.rcsb.org/). Online protein-protein docking analysis between LsrR and CysJ using HDOCK (http://hdock.phys.hust.edu.cn/). (**C**) Online protein-protein docking analysis between LsrR and CysN. Residues within a 5-angstrom range of each protein were identified, and hydrogen bonds were predicted (represented by red dashed lines). Interface residues and bond lengths were listed. 3D protein models and molecular docking results were visualized using PyMOL (version 2.5.7).

Molecular docking was used to predict binding sites between LsrR, CysJ, and CysN. Protein domain information was retrieved from UniProt (https://www.uniprot.org/), confirming the presence of the three proteins and their domains (Fig. 6A). Given the multiple domains in CysJ and CysN, docking analysis revealed specific binding sites. Strong interactions were observed between LsrR and CysJ and CysN (Tables S6 and S7). In the LsrR-CysJ complex, 10 LsrR residues formed hydrogen bonds, primarily in the sugar-binding domain, suggesting its dominant role over the DNA-binding domain (Fig. 6B). A similar trend was found for the LsrR-CysN complex (Fig. 6C). However, the functional implications of these interactions remain unclear.

### LsrR regulates H_2_S production and the oxidative stress response during APEC

**Fig 7:**
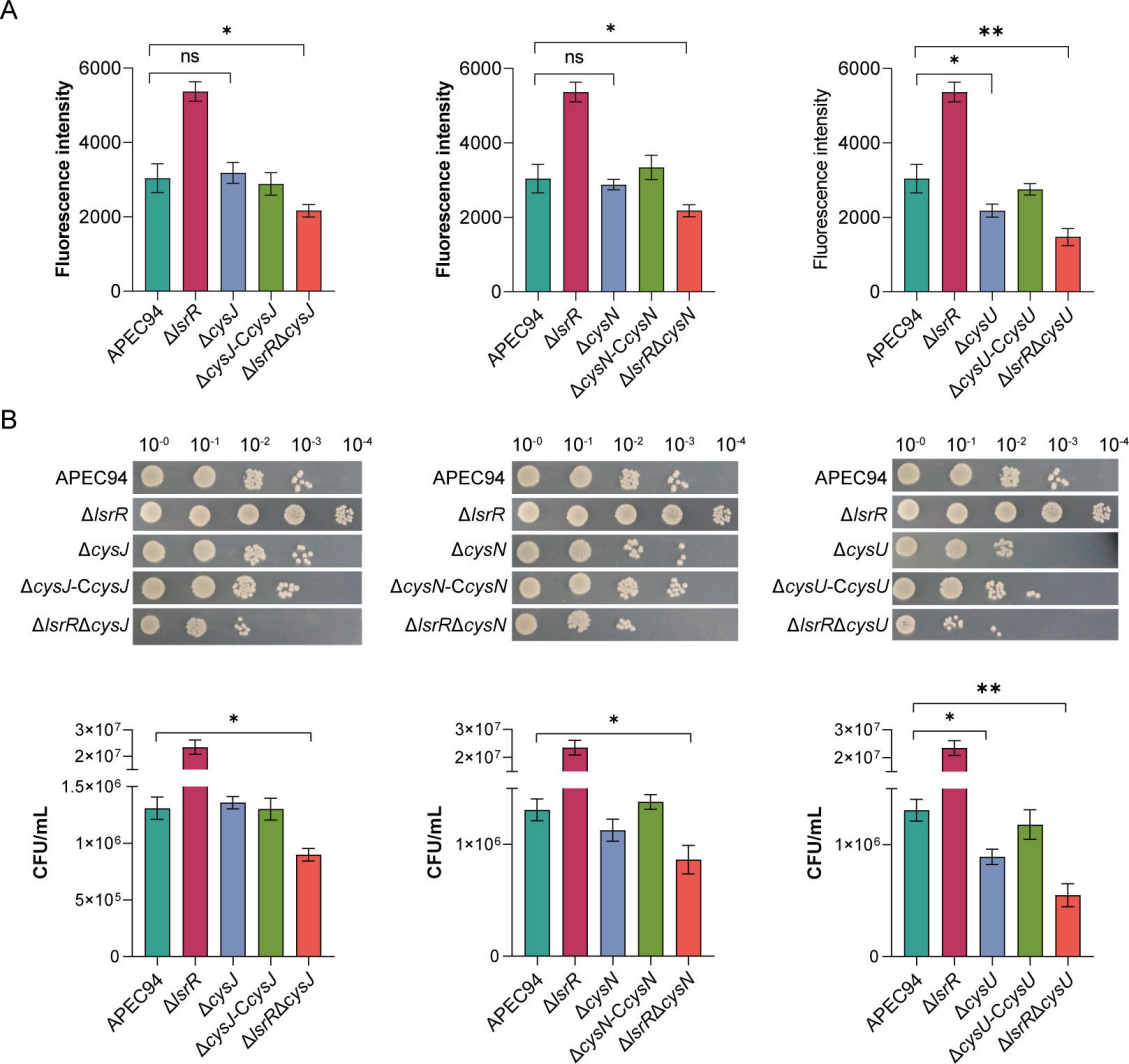
Assessment of H_2_S production and antioxidant stress capacity in *cys* mutant strains. (**A**) Determination of intracellular H_2_S production using the WSP-5 probe. For intracellular H_2_S detection, each strain was cultured to an OD₆₀₀ of 1.0 and supplemented with the WSP-5 probe at a final concentration of 15 μM, followed by incubation at 37°C for 30 min. The bacteria were then resuspended in PBS, and the fluorescence intensity (488–528 nm, FITC channel) was measured using a Cytation3 microplate reader (BioTek). (**B**) Resistance to H_2_O_2_ stress. Each strain (OD_600_ = 1.0) was cultured in 5 mL of LB broth supplemented with 8 μL of 30% H_2_O_2_ and incubated at 37°C for 1 h. Three microliters of each strain was dropped on LB agar plates and incubated at 37°C for 12 h (top panel). The CFUs of each strain were determined using the dilution plating method (bottom panel). Data are presented as the mean ± SD from at least three independent experiments. Student’s *t*-test was used for statistical analysis, with significance indicated as follows: ns (no significant change, *P* > 0.05), *(0.01 < *P* < 0.05). Additionally, the images for “APEC94” and “Δ*lsrR*” are reused across multiple panels. As the experiments for APEC94, Δ*lsrR*, Δ*cysN*, Δ*cysJ*, and Δ*cysU* single and double deletion strains were conducted in the same batch under identical conditions, the shared images were presented in separate subpanels to facilitate clear comparison.

LsrR binds to the *cysPUWAM* promoter and interacts with CysJ and CysN, suggesting its involvement in sulfate assimilation in APEC. To investigate its role in H_2_S production and oxidative stress response, Δ*cysJ*, Δ*cysN*, Δ*cysU*, and Δ*lsrR*-based double-gene mutants were constructed. Deletion of *cysJ* did not affect intracellular H_₂_S levels (*P* > 0.05), but the Δ*lsrR*Δ*cysJ* strain showed a significant decrease in intracellular H_₂_S levels (*P* < 0.05) (Fig. 7A). These results correlated with those of the antioxidative stress assay (Fig. 7B).

Compared with the WT strain, the Δ*lsrR*Δ*cysN* strain presented significantly lower H_2_S levels (*P* < 0.05) (Fig. 7A). Deletion of *lsrR* and *cysN* also impaired the oxidative stress resistance of APEC (*P* < 0.05) (Fig. 7B). Additionally, both the Δ*cysU* and the Δ*lsrR*Δ*cysU* strains showed a significant reduction in H_2_S (*P* < 0.05). However, the deletion of either *cysJ* or *cysN* alone did not significantly affect H_2_S levels (*P* > 0.05) (Fig. 7A). Similarly, compared with the wild type, the Δ*cysJ*Δ*cysN* double mutant did not significantly differ in H_2_S levels (*P* > 0.05) (Fig. S4 and S5). Overall, LsrR plays an inhibitory role in both H_2_S production and the oxidative stress response in APEC.

### *lsrR* deficiency enhances the intracellular survival of APEC in RAW264.7 cells

**Fig 8:**
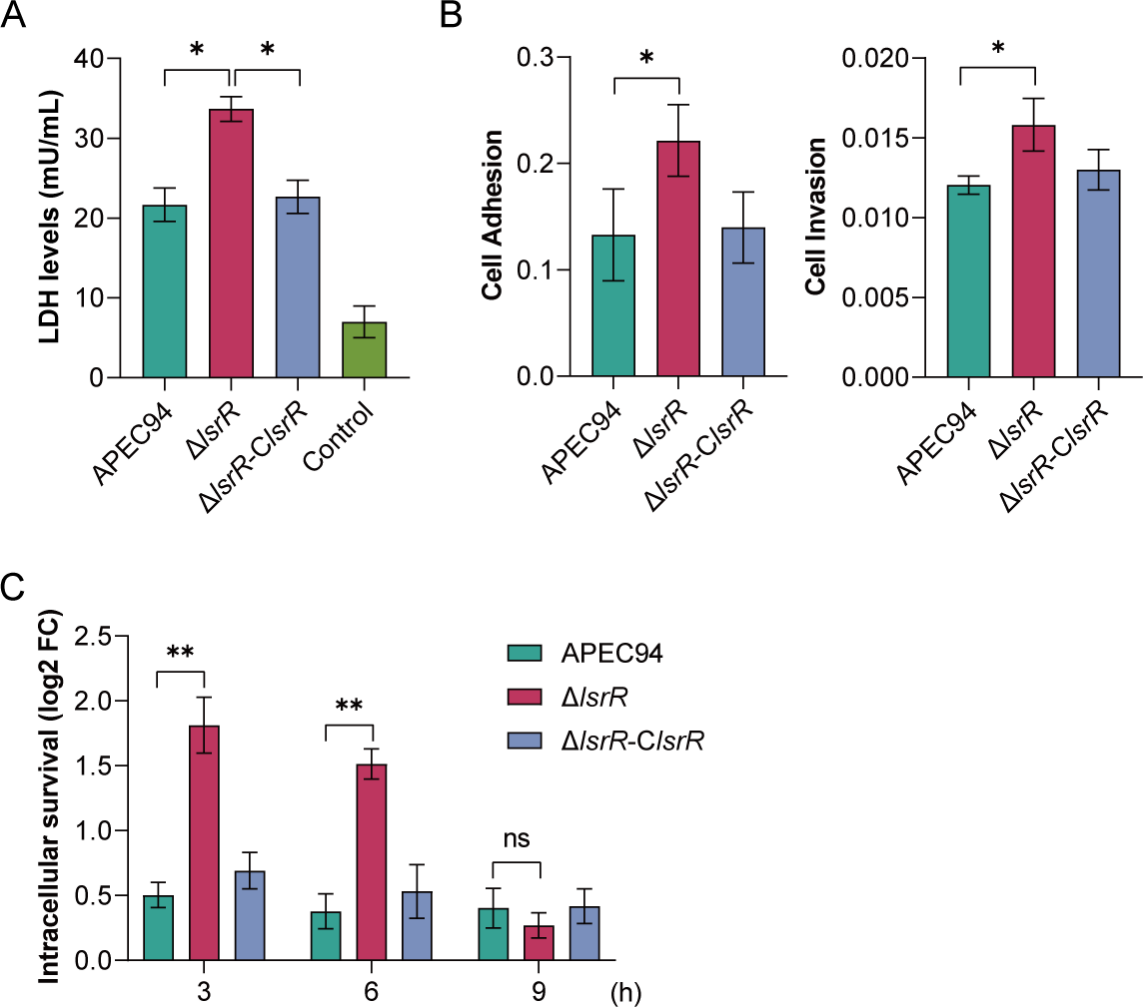
Assessment of LDH release, cell adhesion, invasion, and intracellular survival. (**A**) LDH release assay. RAW264.7 cells were infected with bacterial strains in a 96-well plate for 2 h, and LDH release was quantified separately in the supernatant and the cell lysate. (**B**) Assessment of cell adhesion and invasion. RAW264.7 cells were infected in a 24-well plate at 37°C with 5% CO_2_ for 2 h. The cells were then lysed using 0.5% Triton X-100, and the CFU of each strain was determined using the doubling dilution method. For the invasion assay, after infection under the same conditions, fresh culture medium supplemented with 100 μg/mL gentamicin sulfate was added, and the cells were incubated for 1 h. The cells were then lysed, and the CFU of each strain was determined using the doubling dilution method. (**C**) Measurement of intracellular survival. Fresh culture medium supplemented with 100 μg/mL gentamicin sulfate was added, and the cells were incubated for 3 h, 6 h, and 9 h. At each time point, the cells were lysed using 0.5% Triton X-100, and the CFUs of each strain were determined using the doubling dilution method. Data are presented as the mean ± SD from at least three independent experiments. Student’s *t*-test was used for statistical analysis, with significance indicated as ns (no significant change, *P* > 0.05), *(0.01 < *P* < 0.05), and **(*P* < 0.01).

LsrR regulates sulfate assimilation and the oxidative stress response in APEC, but its role in pathogen-host interactions remains unclear. Lactate dehydrogenase (LDH) release assays revealed that, compared with WT infection, APEC94Δ*lsrR* infection led to a significant increase in LDH levels in RAW264.7 macrophages (*P* < 0.05) (Fig. 8A). Adhesion and invasion assays further revealed that APEC94Δ*lsrR* resulted in significantly increased adhesion and invasion rates in RAW264.7 cells (*P* < 0.05) (Fig. 8B).

Given the role of LsrR in the adhesion and invasion of macrophages, we hypothesize that it influences bacterial survival within macrophages. Intracellular survival assays revealed that, compared with the WT strain, APEC94Δ*lsrR* exhibited significantly greater survival in RAW264.7 macrophages at 3 and 6 h post-infection (*P* < 0.05), but not at 9 h (*P* > 0.05) (Fig. 8C). These findings align with studies showing that *lsrR* deletion increases APEC94 resistance to oxidative stress. Overall, *lsrR* deletion significantly enhances APEC94 adherence, invasion, and intracellular survival in macrophages.

### LsrR regulates nuclear factor-kappa B (NF-κB) signaling during APEC infection

**Fig 9:**
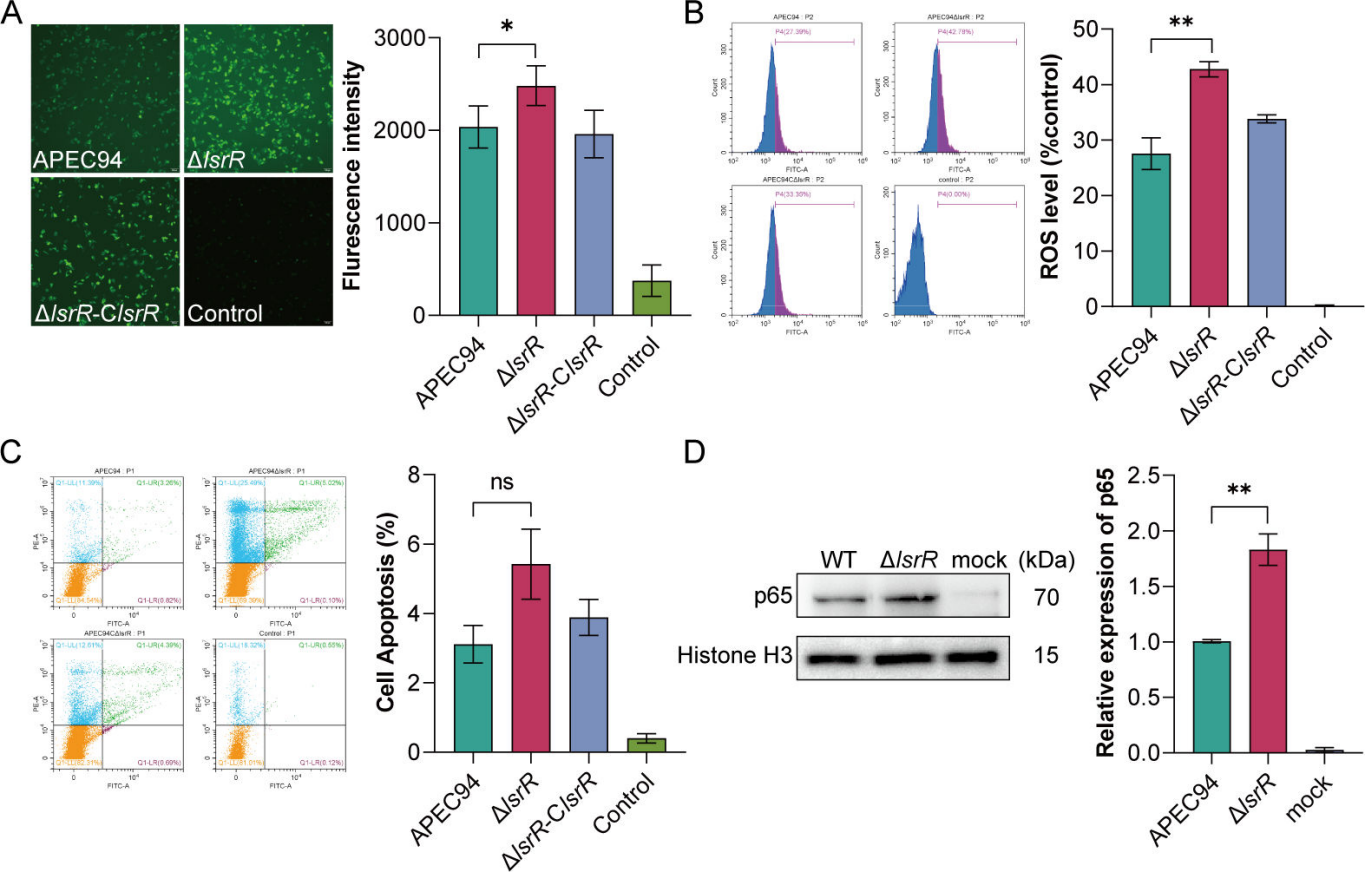
Detection of intracellular reactive oxygen species levels, apoptosis, and nuclear expression of p65. (**A**) Detection of intracellular reactive oxygen species by fluorescence microscopy. RAW264.7 cells were infected for 4 h, followed by treatment with the fluorescent probe DCFH-DA. The fluorescence was observed under a fluorescence microscope, and the data were analyzed using ImageJ software. (**B**) Detection of intracellular reactive oxygen species by flow cytometry. Following a 4 h infection period, RAW264.7 cells were treated with the fluorescent probe DCFH-DA and analyzed by flow cytometry. (**C**) Assessment of apoptosis by flow cytometry. Following a 4 h infection period, RAW264.7 cells were treated with Alexa Fluor 488-12-dUTP and analyzed by flow cytometry. (**D**) NF-κB p65 expression was detected by western blotting. Following a 4 h infection period, RAW264.7 cells were collected, and nuclear proteins were extracted using a nuclear protein extraction kit. The expression of p65 was detected using an anti-p65 monoclonal antibody, while an anti-histone H3 monoclonal antibody was used as a loading control. The data were analyzed using ImageJ software. Data are presented as the mean ± SD from at least three independent experiments. Student’s *t*-test was used for statistical analysis, with significance indicated as ns (no significant change, *P* > 0.05), *(0.01 < *P* < 0.05), and **(*P* < 0.01). “Control” and “Mock” refer to RAW264.7 cells that received the same medium and incubation conditions as the infected groups but were cultured without the addition of any bacteria.

Given the enhanced intracellular survival of the APEC94Δ*lsrR* strain, further investigation of the effects of *lsrR* deletion in macrophages is warranted. Flow cytometry revealed significantly higher ROS levels in RAW264.7 macrophages at 4 h post-infection with the Δ*lsrR* mutant than in those with the WT strain (*P* < 0.05) (Fig. 9A). Fluorescence microscopy confirmed these results (Fig. 9B). However, no significant differences in ROS production were observed at 8 h post-infection (*P* > 0.05) (Fig. S2).

Infection with the Δ*lsrR* strain increased ROS levels in RAW264.7 macrophages, highlighting its role in pathogens-host interactions. To assess APEC-induced apoptosis, the cells were analyzed at 4 h post-infection. No significant difference was observed between the APEC94Δ*lsrR* strain and the WT strain, as determined by Student’s *t*-test (*P* > 0.05) (Fig. 9C).

Proinflammatory cytokines play key roles in early immune responses to infection. RT-qPCR revealed that the transcription of IL-6, IL-12, IL-1β, and TNF-α in macrophages was upregulated approximately onefold upon infection with the APEC94Δ*lsrR* and WT strains (Fig. S3). Western blot analysis confirmed that infection with the Δ*lsrR* strain promoted NF-κB p65 nuclear entry (*P* < 0.05) (Fig. 9D). These findings suggest that *lsrR* inhibits APEC94 adhesion, invasion, and intracellular survival in RAW264.7 macrophages while decreasing NF-κB p65 activation.

## DISCUSSION

The objective of this study was to elucidate the function of *lsrR* in the antioxidative stress response through the regulation of exogenous sulfate assimilation in *E. coli*. Additionally, we observed that deletion of *lsrR* enhanced the resistance of APEC94 to host defense. Our findings indicate that *lsrR* regulates multiple biological processes in *E. coli* and facilitates immune evasion by mediating exogenous sulfate uptake and inhibiting the NF-κB signaling pathway.

This study explored the impact of *lsrR* on intracellular H_2_S levels in *E. coli*, given the role of H_2_S in bacterial resistance to oxidative stress. Deletion of *lsrR* in *E. coli* significantly promoted intracellular H_2_S production (Fig. 2B). *lsrR* expression was reduced by ~3-fold upon treatment with sodium thiosulfate and by ~1-fold upon treatment with sodium sulfide compared with that in the untreated control (Fig. S6). The overexpression of *mstA* similarly enhanced intracellular H_2_S production and oxidative stress resistance while upregulating the transcript levels of *cysW*, *cysA*, *cysD,* and *cysN* by approximately fivefold (exogenous sulfate assimilation) (Table S4). These findings suggest a potential role for sulfate and thiosulfate assimilation in 3-MST-mediated H_2_S resistance to oxidative stress. Our findings revealed that *lsrR* deletion upregulated the transcription of genes involved in sulfate assimilation, including *sbp*, *cysUWA*, *cysDNC*, *cysJIH,* and *cysK*, by approximately ninefold (Table S3). However, the transcript levels of genes involved in thiosulfate assimilation and serine conversion, such as *cysP*, *cysM,* and *cysE*, remained unchanged compared with those in the WT (Table S3). These findings were consistent across the Δ*lsrR* and Δ*lsrR*-C*lsrR* strains, further confirming the primary role of *lsrR* in the exogenous sulfate assimilation pathway (Fig. 3B). In the Δ*mstA* strain, *ycjW* was reported to produce H_2_S by regulating PspE (thiosulfate sulfurtransferase) (21). Moreover, IscS (a cysteine desulfurase) served as the main source of endogenous H_2_S under anaerobic conditions in the Δ*mstA* strain (37). In our study, while *mstA* transcription remained unchanged, *iscS* transcription was upregulated approximately fourfold, whereas *pspE* transcription was downregulated approximately sixfold compared with that in the WT (Table S3), suggesting the involvement of multiple pathways in endogenous H_2_S production.

Deletion of *lsrR* also increased the resistance of APEC94 to oxidative stress (Fig. 2A). The known enzymes KatG and KatE, along with the NADH peroxidase AhpCF, function as catalases and peroxidases that break down H_2_O_2_ in *E. coli* (38, 39). However, in this study, the transcript levels of the *ahpC*, *katG*, and *oxyR* genes in the Δ*lsrR* strain were similar to those in the WT strain. Concurrently, the transcription levels of the *cyuP*, *tcyJ,* and *tcyP* genes were upregulated, whereas that of *ahpF* was downregulated compared with that of the WT (Table S3). This finding aligns with studies showing that *cysK*, *cysP*, and *tcyJ* can be highly regulated in response to H_2_O_2_ independently of *oxyR* (18, 40–42). H_2_S functions as a second messenger in bacteria, enhancing resistance to environmental stresses (43). Notably, the Δ*lsrR* strain exhibited increased intracellular H_2_S production in APEC94 (Fig. 2), supporting the hypothesis that *lsrR*-dependent sulfate transport is an influential factor in *E. coli* resistance to oxidative stress.

This study further explored the regulatory mechanisms involved in *lsrR-*mediated sulfate metabolism. Notably, the regulatory function of LsrR occurs primarily through the presence of a DNA-binding domain that interacts with the gene promoter region. Our findings revealed that LsrR inhibits the transport of exogenous sulfate by directly binding to the promoter region of *cysPUWAM* in APEC94 (Fig. 4D). Deletion of *cysU* resulted in a decrease in intracellular H_2_S levels and a reduction in APEC resistance to H_2_O_2_. In the Δ*lsrR*Δ*cysU* strain, both the H_2_S content and resistance to oxidative stress significantly decreased (Fig. 7), suggesting that the simultaneous deletion of *lsrR* and *cysU* may disrupt other regulatory pathways in APEC94. In the presence of H_2_O_2_ or Cr (VI), the transcriptional levels of genes such as *cysK*, *sbp*, *cysP*, *cysA*, *cysU*, and *cysW* are upregulated, facilitating sulfate translocation and increasing tolerance to environmental stressors (18, 41, 44, 45). Our results indicate that the uptake of exogenous sulfate enhances bacterial resistance to oxidative stress and that *lsrR* inhibits sulfate uptake by mediating *cysPUWAM* transcription, thereby reducing APEC survival in H_2_O_2_.

To further investigate the effect of *lsrR* on H_2_S production, we examined whether the LsrR protein interacts with proteins involved in the L-cysteine synthesis pathway. Our findings revealed that LsrR interacts with CysJ and CysN, which encode the sulfite reductase flavoprotein subunit and sulfate adenylyltransferase subunit 1, respectively, both of which are involved in sulfate assimilation (Fig. 6). In the Δ*lsrR*Δ*cysJ* and Δ*lsrR*Δ*cysN* strains, we observed a significant reduction in H_2_S content and resistance to oxidative stress (Fig. 7). Previous studies in *E. coli* have shown that the Δ*cysJ* and Δ*cysM* strains exhibit decreased bacterial H_2_S production (22). Additionally, when *E. coli* was treated with isobutanol and gamma irradiation, the transcription levels of genes related to the sulfate assimilation pathway, especially *cysIJH*, *cysC*, *cysN*, and *cysWA*, were upregulated, leading to improved tolerance to environmental stress (46, 47). It is well known that the *cysJ* and *cysN* genes play crucial roles in both intracellular H_2_S production and the response to oxidative stress. Our study suggests that LsrR inhibits exogenous sulfate assimilation by interacting with the CysJ and CysN proteins, thereby reducing the resistance of APEC94 to oxidative stress.

Although the interference of LsrR in the sulfate assimilation pathway has been demonstrated, the consequences of this interference on host-pathogen interactions remain to be fully elucidated. The initial phase of infection involves the adhesion and invasion of pathogens into host cells. Notably, the deletion of *lsrR* significantly increased bacterial adhesion and invasion (Fig. 8B). Elevated levels of H_2_S have been shown to increase the resistance of *E. coli*, *Staphylococcus aureus,* and *Vibrio cholerae* to immune defenses, decrease their susceptibility to H_2_O_2_, and promote their colonization within host cells (34, 48). H_2_S was demonstrated to increase the resistance of bacteria to ROS, thereby facilitating their proliferation within host cells. Conversely, exogenous sulfate uptake was reduced in the Δ*sseA* and Δ*cysJ* strains, leading to decreased survival of *E. coli* within the host cells (22, 33). Deletion of *lsrR* increased intracellular H_2_S production by approximately 1.8-fold and enhanced the resistance of APEC94 to macrophage-mediated killing by approximately 29-fold, ultimately promoting a ~4-fold increase in its survival within macrophages. The survival of bacteria within macrophages peaked at approximately 6 h post-infection, followed by a marked decrease at later time points, including 9 h. As the co-culture time increased, the macrophages likely activated a range of intracellular bactericidal mechanisms and experienced nutrient limitation in the intracellular environment, which reduced the survival of intracellular bacteria and ultimately eliminated the initial differences in fitness between the strains. These findings suggest that *lsrR* may potentially decrease bacterial resistance to oxidative stress by inhibiting sulfate assimilation, which in turn affects bacterial viability within host cells.

Host cells improve their resistance to pathogens by modulating immune pathways (49, 50). Cells infected with the *lsrR* mutant strain presented transcriptional levels of proinflammatory cytokines (Fig. S3) and elevated expression of the NF-κB p65 protein (Fig. 9D). Compared with WT, the *∆lsrR* mutant induced a greater increase in intracellular ROS levels upon infection of macrophages (RAW264.7 cells). The loss of *lsrR* may alter bacterial surface characteristics or secretion profiles, enhancing recognition by the host immune system and thereby stimulating stronger oxidative and inflammatory responses. This increase in ROS correlated with the enhanced intracellular survival of the *∆lsrR* mutant observed between 0 and 6 h post-infection. Concurrently, under H_2_O_2_ stress, the deletion of *lsrR* enhances the antioxidant stress response in APEC. These findings highlight the adaptive capacity of the *∆lsrR* mutant and suggest a potential role for LsrR in modulating host immune evasion. The precise regulatory mechanisms underlying this phenomenon remain to be elucidated and warrant further investigation.

**Fig 10:**
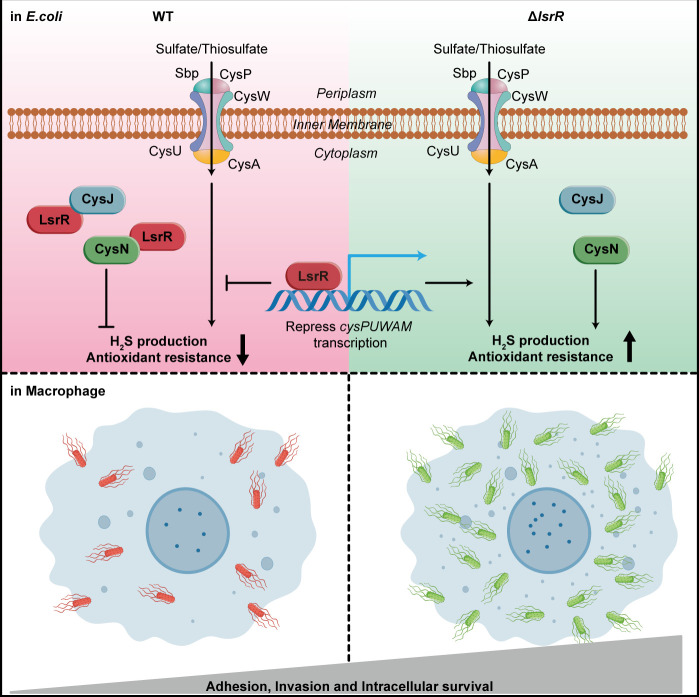
Schematic model of the involvement of LsrR in sulfur metabolism and APEC intracellular survival. LsrR directly represses transcription of the *cysPUWAM* operon and, by interacting with the proteins CysN and CysJ, reduces intracellular H_2_S accumulation and attenuates the bacterial antioxidant response. Additionally, deletion of *lsrR* enhances the adhesion, invasion, and intracellular survival of APEC in macrophages. However, the precise regulatory mechanisms underlying these effects remain to be elucidated.

In conclusion, LsrR represses transcription of the *cysPUWAM* operon and, by interacting with the proteins CysN and CysJ, reduces intracellular H_2_S accumulation and attenuates the bacterial antioxidant response. Additionally, a lack of *lsrR* enhances the intracellular survival of APEC in macrophages (Fig. 10). Given the pivotal role of H_2_S in bacterial antioxidant stress responses, the development of specific inhibitors that target bacterial H_2_S synthesis as novel antibacterial agents is necessary (51, 52). This study highlights the potential value of *lsrR* in enhancing host defense against pathogenic bacterial infection. These findings demonstrate that the upregulation of LsrR expression may serve as an effective strategy to reduce the pathogenicity of APEC, indicating that *lsrR* is a promising target for the prevention and control of APEC infection.

## MATERIALS AND METHODS

### Strains and plasmids

The clinically isolated O_78_ serotype APEC strain APEC94 was stored in the laboratory. The bacteria were cultivated in LB broth at 37°C, and ampicillin (100 μg/mL), chloramphenicol (30 μg/mL), or kanamycin (50 μg/mL) was added when necessary. The plasmids and strains used in this study are listed in Table S1.

### Construction of the *lsrR* mutant and complementary strains

The primers used to knock out lsrR are listed in Table S2. The *lsrR* mutant strain was constructed using the CRISPR-Cas9 system with modifications (53). Upstream and downstream DNA fragments of *lsrR* were amplified. The pTarget-*lsrR*-up-down plasmid was constructed and transformed into APEC94 cells harboring the temperature-sensitive pCas plasmid. pTarget was eliminated with IPTG. For the complementary strain APEC94Δ*lsrR*-C*lsrR*, pSTV28-*lsrR* was electrotransformed into APEC94Δ*lsrR*. Other mutant strains were constructed similarly.

### Assessing biofilm formation with crystal violet staining

Biofilm formation was assessed by crystal violet staining with modifications (54). Each strain was diluted in LB medium and cultured in 96-well polyvinyl chloride microtiter plates at either 25°C or 37°C for 24 h. After they were incubated, the plates were washed, air-dried, and stained with a 1% crystal violet solution for 25 min at 37°C. The crystal violet was dissolved in 95% ethanol, and the absorbance at 595 nm was measured. The experiment was repeated three times.

### Detection of H_2_S content

Extracellular H_2_S detection was carried out using modified lead acetate paper (18). Strips were suspended above the liquid medium for 24 h, after which they captured H_2_S and turned black. Grayscale measurements were used to compare H_2_S production. For intracellular H_2_S, WSP-5 probes (55) were used. The strains were cultured to an OD_600_ of 1.0, supplemented with 15 μM WSP-5 probes (CATB), incubated at 37°C for 30 min, and resuspended in PBS (pH 7.4). The fluorescence intensity at 488–528 nm (FITC) was measured using a Cytation3 (BioTek system). All experiments were conducted in triplicate.

### H_2_O_2_ oxidative stress assay

The overnight-cultured strain was diluted in 5 mL of LB medium and incubated at 37°C for 3 h. Subsequently, 8 μL of 30% H_2_O_2_ was added and incubated at 37°C for 1 h. Colony-forming units (CFUs) of each strain were counted using the doubling dilution method. All the experiments were repeated three times. For clarity and consistency, the images of “APEC94” and “Δ*lsrR*” strains were reused across multiple figure panels. These strains, along with the single- and double-gene deletion strains (Δ*cysN*, Δ*cysJ*, Δ*cysU*), were processed and analyzed under the same experimental conditions and within the same experimental batch. Therefore, the same images were presented in separate subfigures for comparative purposes.

### RNA extraction and RT-qPCR

RNA extraction and RT-qPCR were performed as previously described (56). Bacterial RNA was extracted with TRIzol (Invitrogen), and cDNA was reverse-transcribed using a Vazyme kit. RT-qPCR was performed using a SYBR qPCR kit (Vazyme). mRNA fold changes were calculated by the 2^-ΔΔCt^ method. The housekeeping transcript *dnaE* was used as the internal reference gene to normalize target gene expression. The experiments were repeated three times.

### ChIP-seq

ChIP-seq was performed with modifications (21). At an OD_600_ of 0.4, the strains were incubated at 37°C for 2 h, treated with 1% formaldehyde for 20 min, and quenched with 0.5 M glycine. The cells were collected, washed with Tris-buffered saline, and disrupted by ultrasonication. The resulting DNA fragments were incubated with anti-LsrR antibody in IP buffer (10 mM Tris-HCl [pH 7.4], 0.1% Igepal CA-630, 150 mM NaCl) for 3 h with a DNase inhibitor. The samples were subsequently on the Illumina HiSeq platform.

### EMSA and DNase I footprinting assay

For LsrR purification, BL21(DE3) cells harboring pET-28a-*lsrR* were induced with 0.5 mM IPTG at 28°C for 20 h. After ultrasonic disruption, the supernatant was incubated with Ni-affinity resin (Sigma) at 4°C for 3 h. LsrR was eluted using 500 mM imidazole buffer.

EMSA was performed as previously described with modifications (57). Biotin-modified promoter DNA fragments were incubated with the LsrR protein in binding buffer (100 mM NaCl, 3 mM magnesium acetate, 0.1 mM dithiothreitol, 0.1 mM EDTA, and 50 mM Tris-HCl) at 37°C for 20 min. The protein-DNA complexes were resolved on a 6% nondenaturing gel, transferred to nylon membranes, and detected using horseradish peroxidase (HRP)-labeled streptavidin and ECL substrates. Images were captured using a blot analysis system (Tanon). The experiments were repeated three times.

DNase I footprinting was used to analyze specific binding motifs of *cysPUWAM* and *cysJIH* as previously described with modifications (58). Briefly, biotin-tagged promoter DNA fragments were mixed with the LsrR protein and incubated in binding buffer at 37°C for 20 min. The samples were treated with DNase I (1 U/μL, Thermo Fisher) for 5 min, and the reaction was terminated by phenol–chloroform extraction. DNA was precipitated with ethanol, eluted with ddH_2_O, and sequenced by Sangon Biotech (Shanghai).

### Protein expression and pull-down assay

A GST pull-down assay was performed as previously described with modifications (59). Briefly, BL21(DE3) cells with the pGEX-4T-1-lsrR plasmid were induced with 0.5 mM IPTG at 28°C for 16 h. After 30 min of ultrasonic disruption, the supernatants were collected and incubated with glutathione-tagged magnetic beads (Sigma) at 4°C for 3 h. The protein-bead complexes were subsequently washed three times with PBS. APEC94 whole-cell lysates were added and incubated at 4°C for 12 h. The interaction proteins were eluted with 10 mM GSH and analyzed by LC-MS.

### BACTH assay

The BACTH assay was performed as previously described with modifications (60). The *lsrR* ORF was inserted into the pUT18C plasmid, while the *cysJ* and *cysN* gene ORFs were cloned and inserted into pKT25. Both plasmids were subsequently cotransformed into the BTH101 strain, which was then cultured on LB plates supplemented with 0.5 mM IPTG and 40 μg/mL X-Gal at 30°C. The leucine zipper domain (Zip) of yeast GCN4 served as the positive control, while empty pKT25 or pKT25-Zip plasmids were used as negative controls.

β-Galactosidase activity was measured using a β-Galactosidase Assay Kit (Beyotime) following the manufacturer’s instructions with modifications. BTH101 cells (5 mL, OD_600_ = 1.0) containing both plasmids were harvested and lysed by ultrasonication. The supernatant (20 μL) and 5 μg of o-nitrophenyl-β-D-galactopyranoside were used to detect β-galactosidase activity. The samples were incubated in PBS at 37°C for 20 min, after which the absorbance was measured at 420 nm.

### 3D protein modeling and molecular docking

The protein sequences and structural domain information of LsrR, CysJ, and CysN were obtained from the UniProt database (https://www.uniprot.org/). 3D models of LsrR, CysJ, and CysN were constructed using the SWISS-MODEL server (https://swissmodel.expasy.org/). Online protein-protein docking was then performed between LsrR and CysJ, as well as between LsrR and CysN, using the HDock server (http://hdock.phys.hust.edu.cn/). The resulting protein-protein complexes were visualized using PyMOL open-source software (version 2.5.7). Specifically, residues within a 5-angstrom range of the proteins were selected, and hydrogen bonds were identified and analyzed.

### Infection of RAW264.7 cells

RAW264.7 cells were cultivated in Dulbecco’s modified Eagle’s medium supplemented with 10% fetal bovine serum and infected with APEC (multiplicity of infection [MOI] = 50) in 24-well plates at 37°C with 5% CO_2_ for 2 h, after which the cells were washed with PBS.

For the adhesion assay, cells were lysed with 0.5% Triton X-100, and the CFUs were counted. For the invasion assay, the cells were incubated with 100 μg/mL gentamicin sulfate for 1 h before processing. For the intracellular survival assay, the cells were incubated with gentamicin sulfate for 3, 6, or 9 h, following the same protocol used for the invasion assay.

For the cytotoxicity assay, LDH release was quantified using an LDH Cytotoxicity Assay Kit (Beyotime) following the manufacturer’s protocol. Briefly, RAW264.7 cells were seeded into 96-well plates and coincubated with the respective bacterial strains at a multiplicity of infection (MOI) of 50 for 2 h. Following incubation, the culture supernatants were collected by centrifugation and transferred to a new 96-well plate. Subsequently, 60 μL of LDH assay working solution was added to each well. The plate was gently mixed and incubated at room temperature (25°C) in the dark for 30 min. Absorbance was then measured at 490 nm using a microplate reader.

For western blotting, macrophages were infected in six-well plates (MOI = 50, 4 h) and washed, after which nuclear proteins were extracted using a nuclear protein extraction kit (Beyotime). The samples were denatured at 100°C for 8 min, separated by 12.5% sodium dodecyl sulfate-polyacrylamide gel electrophoresis (80 V for 30 min, 120 V for 40 min), and transferred to methanol-activated PVDF membranes (Merck-Millipore) using a Trans-Blot semidry system (Bio-Rad) at 25 V for 15 min.

The membranes were blocked with 5% skim milk in PBST for 2 h and incubated overnight at 4°C with primary antibodies against p65 (NF-κB subunit) and histone H3 (Cell Signaling), followed by incubation with HRP-labeled anti-IgG antibodies (2 h, RT). The blots were developed with ECL (Beyotime) and analyzed using a Tanon imaging system. The grayscale values were quantified with ImageJ. The experiments were performed in triplicate.

### Flow cytometry and cell sorting

To detect cellular ROS, each strain (MOI = 50) was incubated with RAW264.7 cells in six-well plates for 4 or 8 h at 37°C with 5% CO_2_. After being washed with PBS, 10 μmol/L DCFH-DA probe was added and incubated for 20 min. The cells were harvested, and the ROS concentration was analyzed by flow cytometry (Beckman Coulter), with 10,000 cells per sample. All the experiments were repeated three times.

Apoptosis was assessed using a TUNEL Apoptosis Detection Kit (FITC) (Yease). RAW264.7 cells were infected with each strain for 4 h at 37°C with 5% CO_2_, fixed with 4% paraformaldehyde, and incubated in 70% ethanol. The cells were treated with TdT for 60 min in the dark, and the reaction was stopped with 20 mM EDTA. After resuspension in 0.1% Triton X-100 (containing 5 mg/mL BSA) and collection, the cells were incubated with PI solution in the dark for 30 min. Apoptosis was analyzed by flow cytometry using 10,000 cells per sample. The experiments were performed in triplicate.

### Statistical analysis

Statistical analyses were conducted using GraphPad Prism 8.0 (GraphPad Software Inc., La Jolla, CA, USA). The data are expressed as the mean ± standard error of the mean. Group differences were analyzed using one-way analysis of variance followed by appropriate *post hoc* tests. Statistical significance was set at *P* < 0.05.

## Data Availability

All data included in this study are available upon request by contacting the corresponding author. All RNA-seq data generated in this study have been deposited in the NCBI Gene Expression Omnibus (GEO) under the accession number GSE310657. The ChIP-seq experiments were conducted in 2019, and the original raw FASTQ files are no longer available. Despite extensive attempts to retrieve the raw data, including contacting the former laboratory member who performed the sequencing and requesting archival recovery from the sequencing provider (Novogene), no backups of the raw files remain. The loss of the raw files occurred several years prior to the preparation of this manuscript. Importantly, all key analysis outputs derived directly from the raw ChIP-seq data, including genome-wide peak coordinates, peak intensity metrics, and differential peak comparisons, were preserved and are provided in Data S1–S3 (DOI: https://doi.org/10.5281/zenodo.18366090). These datasets constitute the primary experimental evidence supporting the ChIP-seq results reported in this study and enable independent assessment of peak distribution, motif enrichment patterns, and functional associations at the genome-wide level. To further enhance transparency and biological interpretability, additional downstream analyses were performed in 2025 based exclusively on the preserved ChIP-seq peak datasets. These re-analyses, including peak annotation, differential motif enrichment, and functional enrichment analyses (GO and KEGG), were conducted by the same sequencing and bioinformatics service provider that performed the original ChIP-seq data analysis. The results of these secondary analyses are provided in Data S4–S7 (DOI: https://doi.org/10.5281/zenodo.18366090) and do not involve the generation of any new experimental data. Due to journal limitations on the number of supplementary files, all processed ChIP-seq data supporting this study have been deposited in a public repository with permanent access (Zenodo) under the following DOI: https://doi.org/10.5281/zenodo.18366090. Strict internal data-management procedures have since been implemented to prevent similar data loss in future studies.

## SUPPLEMENTAL MATERIAL

Supplemental figures and tables jb.00044-25-s0001.pdf Figures S1 to S6 and Tables S1 to S7.

ASM does not own the copyrights to Supplemental Material that may be linked to, or accessed through, an article. The authors have granted ASM a non-exclusive, world-wide license to publish the Supplemental Material files. Please contact the corresponding author directly for reuse.
