# Supplementary material for: Quorum-sensing regulator LsrR modulates resistance to oxidative stress by interfering with sulfate assimilation in avian pathogenic Escherichia coli
Source: J Bacteriol. 2026 Jun 12;208(7):e00044-25. doi: 10.1128/jb.00044-25 (PMC13393413; doi:10.1128/jb.00044-25)
Supplement: Supplemental figures and tables — Figures S1 to S6 and Tables S1 to S7. [file jb.00044-25-s0001.pdf]

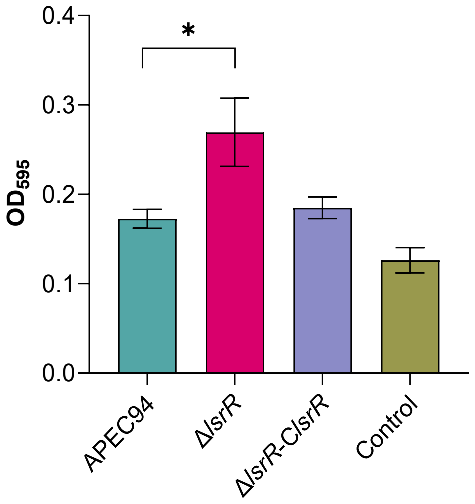

Fig S1 Biofilm detection. *lsrR* mutant and complementary strains were cultured in 96-well polyvinyl chloride (PVC) microtiter plates at 37°C for 24 h. Biofilms were stained with crystal violet, and the bound dye was solubilized in 95% ethanol. The absorbance at 595 nm was measured using a microplate reader. Data are presented as mean ± SD from at least three independent experiments. Student's *t*-test was used for statistical analysis, with significance indicated as ns (no significant change, $P > 0.05$), *($0.01 < P < 0.05$), and **($P < 0.01$).

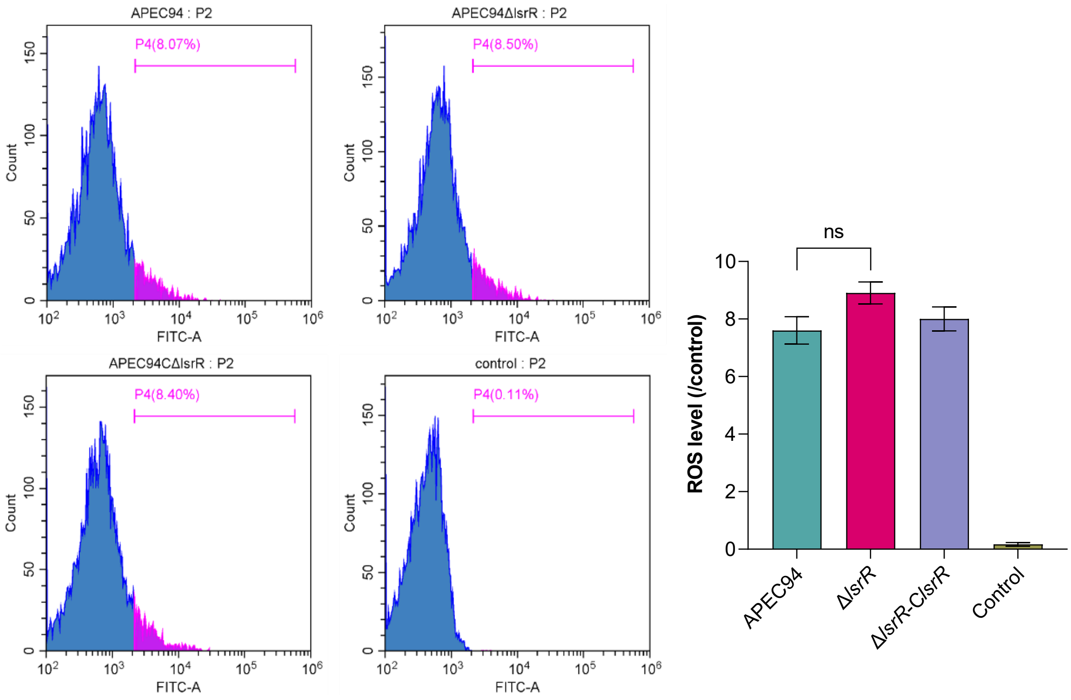

Fig S2 Detection of intracellular reactive oxygen species (ROS) by flow cytometry. Following 8 h period of infection in RAW264.7 cells, the cells were treated with the fluorescent probe DCFH-DA and analyzed by flow cytometry. Data are presented as mean ± SD from at least three independent experiments. Student's t-test was used for statistical analysis, with significance indicated as ns (no significant change, $P > 0.05$), *($0.01 < P < 0.05$), and **($P < 0.01$).

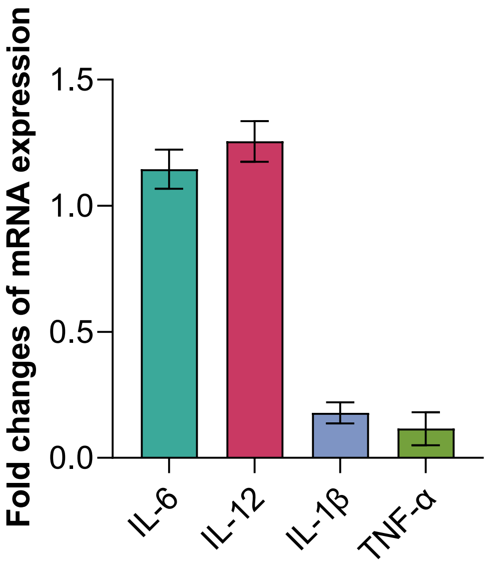

Fig S3 Measurement of cytokine-related genes transcription levels. The relative mRNA levels of cytokine-related genes were measured in APEC94 and APEC94Δ*lsrR*. After 4 h of infection in RAW264.7 cells, total RNA was extracted from cell samples using TRIzol, and relative mRNA levels were quantified by real-time PCR. Data are presented as mean ± SD from at least three independent experiments.

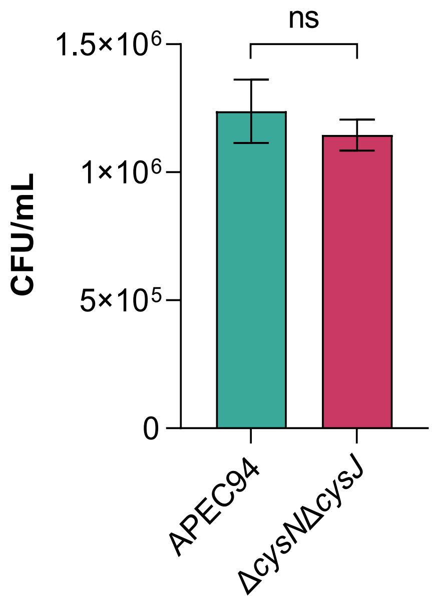

Fig S4 Resistance to H_2O_2. Wild-type, Δ*cysN*Δ*cysJ* mutant, and complementary strains (OD_{600} = 1.0) were cultured in 5 mL LB broth supplemented with 8 μL of H_2O_2 (30%) at 37 °C for 1 h. Colony-forming units (CFU) of each strain were counted using the dilution method.(no significant change, *P* >0.05), *(0.01<*P* < 0.05), and **(*P* < 0.01).

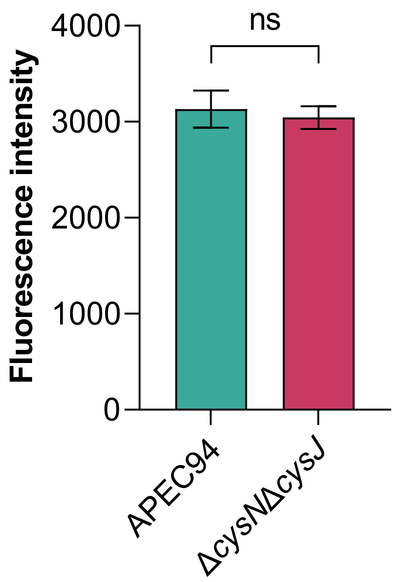

Fig S5 For intracellular H_2S detection, each strain was cultured (OD_{600}=1.0) and supplemented with WSP-5 probe at a final concentration of 15 μM, then incubated at 37°C for 30 min. Bacteria were resuspended in PBS, and fluorescence intensity at 488 ~528 nm (FITC) was measured using the Cytation3 (Biotek).(no significant change, $P > 0.05$), *($0.01 < P < 0.05$), and **($P < 0.01$).

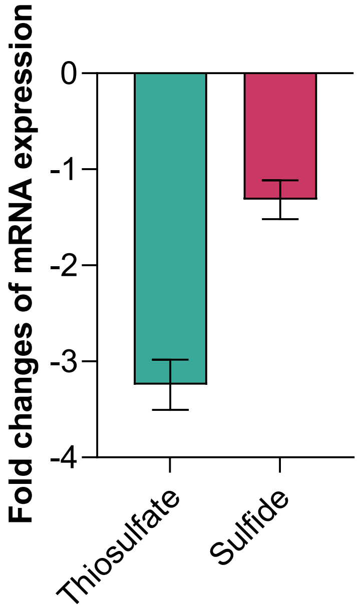

Fig S6 The mRNA levels of *lsrR* in the wild-type strain under different culture conditions were analyzed by reverse transcription quantitative PCR (RT-qPCR). Total RNA was extracted using TRIzol reagent from wild-type cells cultured in LB medium supplemented with either sodium thiosulfate or sodium sulfide. Real-time qPCR was performed using the SYBR qPCR Kit, and the $2^{-\Delta\Delta Ct}$ method was used to calculate fold change. Data are presented as mean ± SD from at least three independent experiments.

Supplementary Table S1. Strains and plasmids used in this study

Strains and plasmids	Description	Source or ref.
Strains		
APEC94	APEC94 O_{78} serotype wild strain	Stored in laboratory
APEC94Δ*lsrR*	*lsrR* gene mutant strain based on APEC94	Stored in laboratory
APEC94Δ*lsrR*-C*lsrR*	*lsrR* gene complementary strain based on APEC94Δ*lsrR*	Stored in laboratory
APEC94Δ*cysU*	*cysU* gene mutant strain based on APEC94	This study
APEC94Δ*lsrR*Δ*cysU*	*cysU* gene mutant strain based on APEC94Δ*lsrR*	This study
APEC94Δ*cysJ*	*cysJ* gene mutant strain based on APEC94	This study
APEC94Δ*lsrR*Δ*cysJ*	*cysJ* gene mutant strain based on APEC94Δ*lsrR*	This study
APEC94Δ*cysN*	*cysN* gene mutant strain based on APEC94	This study
APEC94Δ*lsrR*Δ*cysN*	*cysN* gene mutant strain based on APEC94Δ*lsrR*	This study
BTH101	BACTH assay reporter strain	Stored in laboratory
BL21(DE3)	Prokaryotic expression system strain contained λDE3 prophage carrying the T7 RNA polymerase gene	Stored in laboratory
DH5α	Highly efficient competent cells for plasmid construction	Stored in laboratory
Plasmids		
pTargeT	Spec; constitutive expression of sgRNA without donor editing template DNA	Stored in laboratory
pCas	Kan; expresses Cas9 plus an HDV ribozyme-sgRNA for genome editing	Stored in laboratory
pSTV28-*lsrR*	Cm; low copy plasmid for expressing *lsrR* in APEC94Δ*lsrR*	Stored in laboratory
pGEX-4T-1-*lsrR*	Amp; high copy plasmid with Tac promoters for expressing N-GST induced by IPTG	This study
pET-28a-*lsrR*	Kan; high copy plasmid with T7 and lac promoters for expressing His-tagged LsrR induced by IPTG	This study
pUT18c-*lsrR*	Amp; high copy plasmid with ColE1 ori for expressing 18T-LsrR	This study
pKT25-*cysN*	Kan; low copy plasmid with p15A ori for expressing 25T-CysN	This study
pKT25-*cysJ*	Kan; low copy plasmid with p15A ori for expressing 25T-CysJ	This study

Supplementary Table S2. Primers used in this study

Primers	Sequences (5'-3')	Target genes
Primers for *cysU* mutant and complementary strain construction		
cysU-sgRNA-F	AATACTAGTCTTTACCAGCATTCCGTTTGGTTTTAGAGCTAGAA	*cysU* sgRNA for pTargeT
cysU-sgRNA-R	TTCACTTCCGATTCAAAAAAAGCACCGACTCG	
cysU-UF	TTTTTTGAATCGGAAGTGAACAACATCCGTAAAC	*cysU* upstream DNA
cysU-UR	TTCCGCCATTTCAGTTACGCCCCGCCGCTAACAG	
cysU-DF	GCGTAACTGAAATGGCGGAAGTGACACAATTGAAG	*cysU* downstream DNA
cysU-DR	CCAAGCTTCCGTTAGAGCCGTAGAACAGCAAA	
cysU-inF	GCCTACAAAGTAACGCTGCTGTC	Inside of *cysU*
cysU-inR	CGTTTCCGCCGCTTCTTCATATTC	
cysU-OF	ATGGGGCGCAGCGGATAAAGCT	Outside of *cysU*
cysU-OR	CATAATTTGCAGGTTATGCTCGTCG	
pSTV28-*cysU*-F	GAAAGCTTATGTTTGCTGTCTCCTCCAG	for pSTV28-*cysU*
pSTV28-*cysU*-R	AAGAATTCTTAATGACCTACCACACGCC	
Primers for *cysN* mutant and complementary strain construction		
cysN-sgRNA-F	AATACTAGTTCCAACACTGCTCGAAGTGCGTTTTAGAGCTAGAA	*cysN* sgRNA for pTargeT
cysN-sgRNA-R	TTTGTTAATTATTCAAAAAAAGCACCGACTCG	
cysN-UF	TTTTTTGAATAATTAACAAAGGCGAAAGCATCCGC	*cysN* upstream DNA
cysN-UR	CAGCGCCATTTCTTAAAAATACCCCTGACGTTTTTTCAG	
cysN-DF	ATTTTTAAGA AATGGCGCTGCATGACGAAAACGT	*cysN* downstream DNA
cysN-DR	CCAAGCTT ACAAACACTTCAATAAAGCGCCCTTCT	
cysN-inF	ATCAGCTCTCATCGCTGCATAACGAC	Inside of *cysN*
cysN-inR	GATCCACCAGGCCGATGCCGTTCA	
cysN-OF	TACGCTGGGCTGCTGGCCGCTGAC	Outside of *cysN*
cysN-OR	CCAGCAGATACGTACTGACGCCGA	
pSTV28-*cysN*-F	GAAAGCTTATGAACACCGCACTTGCACA	for pSTV28-*cysN*
pSTV28-*cysN*-R	AAGAATTCTTATTTATCCCCCAGCAAAT	
Primers for *cysJ* mutant and complementary strain construction		
cysJ-sgRNA-F	AATACTAGTTGGCAGCGCTACGTCAAAGAGTTTTAGAGCTAGAA	*cysJ* sgRNA for pTargeT
cysJ-sgRNA-R	TCGAAGCCGCATTCAAAAAAAGCACCGACTCG	
cysJ-UF	TTTTTTGAATGCGGCTTCGAAGGTGAAATCTTT	*cysJ* upstream DNA
cysJ-UR	TTCGCTCATTTGCGTCGTTATGTTCCAGTAAGC	
cysJ-DF	TAACGACGCAAATGAGCGAAAAACATCCAGGGCCT	*cysJ* downstream DNA
cysJ-DR	CCAAGCTTTGGTCAGGCGAATGCTGCCATAGA	
cysJ-inF	GGTATAACTATTATCTCCGCCTCGCA	Inside of *cysJ*
cysJ-inR	TAGCGTCGCCGCAGACATAAATGTG	
cysJ-OF	CCATAAAGGAATGCCCGTGCA	Outside of *cysJ*
cysJ-OR	TCTTTTTCAGAATGCCGTGGAACT	
pSTV28-*cysJ*-F	GAAAGCTTATGACGACACAGGTCCCACC	for pSTV28-*cysJ*
pSTV28-*cysJ*-R	AAGAATTCTTAGTAGACATCTCGCTGAT	
Primers for expression of *LsrR* protein		

Table S2 countine		
4T-1-*lsrR*-F	CGGAATTCATGACAATCAACGATTCGGC	*lsrR* ORF for pGEX-4T-1
4T-1-*lsrR*-R	GCGTCGACTTAACTACGTAAAATCGCCGCT	
28a-*lsrR*-F	GGGGTACCATGACAATCAACGATTCGGC	*lsrR* ORF for pET-28a
28a-*lsrR*-R	CGGGATCCTTAACTACGTAAAATCGCCGCT	
Primers for BACTH		
lsrR-25F	GAGGATCCCATGACAATCAACGATTCGGC	*lsrR* ORF for pKT25
lsrR-25R	AAGGGTACCTTAACTACGTAAAATCGCCGCTG	
cysJ-18F	GCCAAGCTTCATGACGACACAGGTCCCACC	*cysJ* ORF for pUT18c
cysJ-18R	TCGAATTCTTAGTAGACATCTCGCTGATAACGG	
cysN-18F	GCCAAGCTTCATGGTTGATACAACTCAAAC	*cysN* ORF for pUT18c
cysN-18R	TCGAATTCCAGTCCCAGCAGGCCGCAAG	
cysP-18F	GCCAAGCTTCATGGCCGTTAACTTACTGAAAAA	*cysP* ORF for pUT18c
cysP-18R	TCGAATTCTCAGTTACGCCCCGCCGC	
cysU-18F	GCCAAGCTTCATGTTTGCTGTCTCCTCCAGA	*cysU* ORF for pUT18c
cysU-18R	TCGAATTCTTAATGACCTACCACACGCCG	
cysW-18F	GCCAAGCTTCATGGCGGAAGTGACACAATT	*cysW* ORF for pUT18c
cysW-18R	TCGAATTCTCAATGCTCATGATGTTCCTCCT	
cysA-18F	GCCAAGCTTCATGAGCATTGAGATTGCCAAT	*cysA* ORF for pUT18c
cysA-18R	TCGAATTCTCAGGCGCTTTGTGCGAG	
cysD-18F	GCCAAGCTTCATGGATCAAATACGACTTACTCACC	*cysD* ORF for pUT18c
cysD-18R	TCGAATTCTTAAAAATACCCCTGACGTTTTTTC	
cysC-18F	GCCAAGCTTCATGGCGCTGCATGACGA	*cysC* ORF for pUT18c
cysC-18R	TCGAATTCTCAGGATCTGATAATATCGTTCTGTCT	
cysH-18F	GCCAAGCTTCATGTCCAAACTCGATCTAAACGC	*cysH* ORF for pUT18c
cysH-18R	TCGAATTCTTACCCTTCGTGCAATCCACA	
cysI-18F	GCCAAGCTTCATGAGCGAAAAACATCCAGG	*cysI* ORF for pUT18c
*cysI*18R	TCGAATTCTTAATCCCACAAATCGCGCG	
cysK-18F	GCCAAGCTTCATGAGTAAGATTTTTGAAGATAACTCG	*cysK* ORF for pUT18c
cysK-18R	TCGAATTCTTACTGTTGCAGTTCTTTCTCAGTGA	
cysM-18F	GCCAAGCTTCGTGAGTACATTAGAACAAACAATAGGC	*cysM* ORF for pUT18c
cysM-18R	TCGAATTCTTAAATCCCCGCCCCCTG	
cysE-18F	GCCAAGCTTCATGTCGTGTGAAGAACTGGAAA	*cysE* ORF for pUT18c
cysE-18R	TCGAATTCTTAGATCCCATCCCCATACTCAA	
Sbp-18F	GCCAAGCTTCATGAACAAGTGGGGCGTAGG	*Sbp* ORF for pUT18c
Sbp-18R	TCGAATTCTCAGCGTTTGCTGATCTGATC	
tcyP-18F	GCCAAGCTTCATGAACTTTCCATTAATTGCGAA	*tcyP* ORF for pUT18c
tcyP-18R	TCGAATTCTTAACGGTGTGCCAGTTCGG	
tcyJ-18F	GCCAAGCTTCATGAAATTAGCACATCTGGGACG	*tcyJ* ORF for pUT18c
tcyJ-18R	TCGAATTCTTATTTGGTCACATCAGCACCAA	
tcyL-18F	GCCAAGCTTCATGCAAGAAAGTATACAACTGGTTATT	*tcyL* ORF for pUT18c

Table S2 countine		
tcyL-18R	TCGAATTCTCATTTTGGTTCTCTCTCCTGGC	
tcyN-18F	GCCAAGCTTCATGAGTGCCATTGAAGTTAAGAAC	*tcyN* ORF for pUT18c
tcyN-18R	TCGAATTCTTATTGCAGCAGAAACTTCTCAAGG	
cyuP-18F	GCCAAGCTTCATGGAAATTGCATCGAATAAAGG	*cyuP* ORF for pUT18c
cyuP-18R	TCGAATTCTCAAGAAAATGCCAGGAACGG	
cyuR-18F	GCCAAGCTTCATGTTAGATAAAATTGACCGTAAGCT	*cyuR* ORF for pUT18c
cyuR-18R	TCGAATTCTTATTCGATGGGTAAAGAAGTGGTG	
Primers for RT-qPCR		
rt*dnaE*-F	AAAGTTAAACCGGCGAGTGC	*dnaE*
rt*dnaE*-R	TGCCGCTTTTAACGCATCAC	
rt*Sbp*-F	ATGATCCAACGCGCGAATTG	*Sbp*
rt*Sbp*-R	TCAGTACATCGCCAATCCCG	
rt*cysP*-F	AACATTCTGGCGGAATTCCC	*cysP*
rt*cysP*-R	TAGGCTTTGGCGGCTTTTTC	
rt*cysU*-F	GTCTGCTGTTTGTGTGCCTG	*cysU*
rt*cysU*-R	GCAGCACCACTTTGCAGAAA	
rt*cysW*-F	TGGTATCGGCGTTCATCCTG	*cysW*
rt*cysW*-R	TAATGTGACGCGACGGAACA	
rt*cysA*-F	ATGGTGCAGCTTGCTCATCT	*cysA*
rt*cysA*-R	GGGTGTAGTGACCTTTCGGG	
rt*cysC*-F	CGCTGCATGACGAAAACGTC	*cysC*
rt*cysC*-R	GGCGCTTCGTAAACGGAATC	
rt*cysD*-F	CATGTCGATACCGGCTGGAA	*cysD*
rt*cysD*-R	GCAGGTCGATACGGTTGTCA	
rt*cysN*-F	CGTGGCTTCGCAAAGTGAAA	*cysN*
rt*cysN*-R	GCAGGTTTTCAACTTCGCGT	
rt*cysH*-F	GACACCGGCTATCTGTTCCC	*cysH*
rt*cysH*-R	ACCCTTCGTGCAATCCACAT	
rt*cysI*-F	CTACCTGCCGCGTAAGTTCA	*cysI*
rt*cysI*-R	GATCTTCGCGATCTCCAGCA	
rt*cysJ*-F	AACGCCGCTCCTTAGTAACA	*cysJ*
rt*cysJ*-R	TGCGTTATCCATCTGTTCCA	
rt*cysK*-F	CGGTGCCAACATGATTTGGG	*cysK*
rt*cysK*-R	ATCTCTTCACCAGCCAGTGC	
rt*cysM*-F	AAAGATCGTGCGGCACTTTC	*cysM*
rt*cysM*-R	TTTCAGCGCGGCAATCATTG	
rt*cysE*-F	TCGCGCATTGGCAATCTTTC	*cysE*
rt*cysE*-R	ATGCCACGGCCAATTTTTGC	
rt*IL-6*-F	TAGTCCTTCCTACCCCAATTTCC	*IL-6*

Table S2 countine		
rt*IL-6*-R	TTGGTCCTTAGCCACTCCTTC	
rt*IL-12*-F	CCCTTGCCCTCCTAAACCAC	*IL-12*
rt*IL-12*-R	AAGGAACCCTTAGAGTGCTTACT	
rt*IL-1β*-F	GCAACTGTTCCTGAACTCAACT	*IL-1β*
rt*IL-1β*-R	ATCTTTTGGGGTCCGTCAACT	
rt*TNF-α*-F	GACGTGGAACTGGCAGAAGAG	*TNF-α*
rt*TNF-α*-R	TTGGTGGTTTGTGAGTGTGAG	

Note: underline indicates restriction endonuclease sites

Supplementary Table S3. Gene expression profiles from APEC94Δ*lsrR* and WT strains were generated by transcriptome sequencing. Changes in gene expression equal to or greater than 1-fold with p values of at least 0.05 are shown.

Number	Gene name	Fold change	P value	Function
APECO78_RS00340	*sbp*	2.0424 up	2.40E-06	Sulfate/thiosulfate ABC transporter periplasmic binding protein
/	*cysP*	/	/	Thiosulfate/sulfate ABC transporter periplasmic binding protein
APECO78_RS16525	*cysU*	3.4326 up	4.55E-30	Sulfate/thiosulfate ABC transporter inner membrane subunit
APECO78_RS16520	*cysW*	6.8991 up	1.72E-41	Sulfate ABC transporter permease
APECO78_RS16515	*cysA*	9.8676 up	1.9E-102	Sulfate/thiosulfate import ATP-binding protein *cysA*
APECO78_RS18090	*cysC*	6.1997 up	7.91E-21	Adenylyl-sulfate kinase
APECO78_RS18100	*cysD*	2.0321 up	1.47E-23	Sulfate adenylyltransferase subunit 2
APECO78_RS18095	*cysN*	5.6291 up	1.59E-173	Sulfate adenylyltransferase
APECO78_RS18160	*cysH*	6.1411 up	1.32E-46	Phosphoadenosine phosphosulfate reductase
APECO78_RS18165	*cysI*	5.4642 up	8.67E-93	Sulfite reductase subunit beta
APECO78_RS18170	*cysJ*	3.6258 up	6.46E-48	Sulfite reductase subunit alpha
APECO78_RS16480	*cysK*	2.0822 up	9.89E-138	Cysteine synthase A
/	*cysM*	/	/	Cysteine synthase B
/	*cysE*	/	/	Serine acetyltransferase
/	*cysB*	/	/	DNA-binding transcriptional dual regulator CysB
/	*ahpC*	/	/	Alkyl hydroperoxide reductase
APECO78_RS06815	*ahpF*	2.0930 down	4.57E-14	Alkyl hydroperoxide reductase subunit F
/	*mstA*	/	/	3-mercaptopyruvate sulfurtransferase
/	*oxyR*	/	/	DNA-binding transcriptional dual regulator OxyR
APECO78_RS17055	*iscS*	3.8389 up	1.04E-155	IscS subfamily cysteine desulfurase
APECO78_RS13100	*pspE*	6.1067 down	7.58E-23	thiosulfate sulfurtransferase
APECO78_RS20180	*cyuP*	6.8182 up	9.42E-32	L-cysteine importer

Table S3 countine				
/	*cyuR*	/	/	NA-binding transcriptional activator DecR
APECO78_RS13920	*tcyJ*	2.2615 up	1.31E-115	cystine ABC transporter substrate-binding protein
APECO78_RS12955	*tcyP*	2.4937 up	7.38E-16	L-cystine transporter TcyP
/	*tcyL*	/	/	cystine ABC transporter membrane subunit
/	*tcyN*	/	/	cystine ABC transporter ATP binding subunit

Note: Forward slash indicate data that are not present in the transcriptome.

Supplementary Table S4. The data in this table are referenced from the transcriptome sequencing of this article(1). Gene expression profiles from *mstA*-overexpressing and WT strains were generated via transcriptome sequencing.

**Number**	**Gene name**	**Fold change**	**P value**	**Function**
BW25113_RS14320	*cysD*	6.9784	0.010375712	sulfate adenylyltransferase subunit CysD
BW25113_RS12640	*cysW*	5.5772	0.033850115	sulfate/thiosulfate ABC transporter permease CysW
BW25113_RS12635	*cysA*	5.4326	0.023604836	sulfate/thiosulfate ABC transporter ATP-binding protein CysA
BW25113_RS14315	*cysN*	3.8109	0.025073773	sulfate adenylyltransferase subunit CysN

References

1. Nonoyama S, Maeno S, Gotoh Y, Sugimoto R, Tanaka K, Hayashi T, Masuda S. 2024. Increased intracellular H_2S levels enhance iron uptake in *Escherichia coli*. MBio 15:e0199124.

Supplementary Table S5. Proteins identified by LC-MS/MS in GST pull-down samples expressing LsrR.

ProteinID	Protein Name	Gene Name	Coverage [%]	#Unique Peptides	#PSMs	Abundance (Grouped): LsrR	GO_Term	Biological Process (BP)	Molecular Function (MF)	Description
B1XCS7	Sulfate adenylyltransferase subunit 1	*cysN*	3	2	2	1098835	hydrogen sulfide biosynthetic process [GO:0070814]; sulfate assimilation [GO:0000103]; GTP binding [GO:0005525]; ATP binding [GO:0005524]; sulfate adenylyltransferase (ATP) activity [GO:0004781]; GTPase activity [GO:0003924]	hydrogen sulfide biosynthetic process [GO:0070814]; sulfate assimilation [GO:0000103]	GTP binding [GO:0005525]; ATP binding [GO:0005524]; sulfate adenylyltransferase (ATP) activity [GO:0004781]; GTPase activity [GO:0003924]	Sulfate adenylyltransferase subunit 1 OS=Escherichia coli (strain K12 / DH10B) OX=316385 GN=*cysN* PE=3 SV=1
A0A6D2WVG7	Sulfite reductase [NADPH] flavoprotein alpha-component	*cysJ*	3	2	2	942294.0625	hydrogen sulfide biosynthetic process [GO:0070814]; cysteine biosynthetic process [GO:0019344]; sulfate assimilation [GO:0000103]; flavin adenine dinucleotide binding [GO:0050660]; FMN binding [GO:0010181]; sulfite reductase (NADPH) activity [GO:0004783]	hydrogen sulfide biosynthetic process [GO:0070814]; cysteine biosynthetic process [GO:0019344]; sulfate assimilation [GO:0000103]	flavin adenine dinucleotide binding [GO:0050660]; FMN binding [GO:0010181]; sulfite reductase (NADPH) activity [GO:0004783]	Sulfite reductase [NADPH] flavoprotein alpha-component OS=Escherichia coli (strain K12) OX=83333 GN=cysJ PE=3 SV=1

Supplementary Table S6. Molecular docking predicts the interaction sites between LsrR and CysJ

LsrR	CysJ
E17	A49
R21	A50
N65	A209
Q115	A219
R88	A88
H86	D84
Q85, L84	R198
R80	E196
R79, L84	S195
D254	K307

Supplementary Table S7. Molecular docking predicts the interaction sites between LsrR and CysN

LsrR	CysN
S9	R365
Q11	S354, R365
C14	D356
E16	N426
Q63	T309
H28	D317
I59	R320
Q117	V97
Q118	Y101
N189, N191	R68
K194	D65
D195	R89
Q201	D57, Q58
K274	Y55
T275	Q58
